# Diet, hormones, and breast cancer: biomarker-guided assessment of risk and prognosis

**DOI:** 10.3389/fimmu.2026.1946846

**Published:** 2026-09-29

**Authors:** Jia Liu, Mohan Liu, Xiaoying Zhong, Qiang Sun, Xingtong Zhou

**Affiliations:** Department of Breast Surgery, Peking Union Medical College Hospital, Chinese Academy of Medical Sciences & Peking Union Medical College, Beijing, China

**Keywords:** breast cancer, endocrine signaling, metabolism, metabolomic signatures, nutritional biomarkers, precision nutrition, prognosis, risk stratification

## Abstract

Despite growing recognition that nutritional factors modulate breast cancer risk and prognosis through endocrine and metabolic pathways, existing reviews have predominantly focused on individual nutrients or single hormonal axes, lacking an integrated framework that systematically connects nutritional biomarkers across biological scales to clinical prediction models. Furthermore, the translational gap between biomarker discovery and clinical implementation—compounded by pre-analytical variability, population heterogeneity, and the absence of standardized testing protocols—remains inadequately addressed in the current literature. This review synthesizes contemporary evidence on nutritional biomarkers and their interactions with endocrine-related mechanisms in breast cancer, with a specific focus on risk assessment and prognosis prediction. We systematically integrate four core endocrine-metabolic axes (estrogen signaling, insulin/IGF-1 pathway, adipokine homeostasis, and inflammatory-oxidative stress) with four functional classes of nutritional biomarkers (recovery, concentration, replacement, and predictive), delineating their hierarchical relationships and complementary clinical roles. We further examine subtype-specific metabolomic signatures associated with differential prognosis across triple-negative, luminal-like, and HER2-enriched breast cancer, and evaluate the clinical utility of integrating multi-dimensional biomarkers into prognostic prediction models including nomograms. Our analysis reveals that while circulating vitamins, fatty acid profiles, and inflammatory biomarkers demonstrate consistent associations with breast cancer risk, their prognostic value varies substantially by molecular subtype and menopausal status; composite metabolomic signatures outperform single biomarkers in prognostic discrimination, yet most evidence derives from retrospective single-center cohorts with limited external validation. We conclude that biomarker-guided precision nutrition holds substantial promise for advancing personalized breast cancer prevention and management, but critical barriers—including non-standardized assay protocols, underrepresentation of non-Western populations, and a paucity of prospective intervention trials—must be addressed before widespread clinical translation.

## 1 Introduction

Breast cancer remains the most frequently diagnosed malignancy among women worldwide and continues to represent one of the leading causes of cancer-related mortality despite substantial advances in screening strategies, molecular classification, targeted therapies, and multidisciplinary management. The incidence of breast cancer continues to rise across both developed and developing countries, largely driven by population aging, urbanization, obesity, westernized dietary habits, delayed childbearing, and lifestyle transitions. Consequently, breast cancer has become not only a major clinical challenge but also an increasingly important global public health issue that imposes enormous medical, psychological, and socioeconomic burdens. Although remarkable improvements in precision oncology have significantly prolonged survival for many patients, disease heterogeneity, therapeutic resistance, tumor recurrence, and metastatic progression remain formidable barriers to achieving durable clinical benefits. These challenges have shifted increasing attention from treatment alone toward strategies emphasizing primary prevention, individualized risk prediction, and early intervention (1). Growing evidence (2, 3) highlights the pivotal role of nutrition and endocrine factors in modulating breast cancer risk and disease progression. The complex interplay between dietary patterns, metabolic health, and hormonal signaling pathways offers a fertile ground for developing novel approaches to breast cancer control (4).

Unlike inherited genetic susceptibility, many nutritional and metabolic exposures are potentially modifiable throughout life, providing unique opportunities for preventive intervention. Dietary components influence virtually every stage of breast carcinogenesis, including tumor initiation, clonal expansion, metabolic adaptation, immune surveillance, angiogenesis, and metastatic dissemination. Rather than acting through isolated mechanisms, nutritional factors orchestrate extensive biological networks involving systemic metabolism, endocrine homeostasis, chronic inflammation, oxidative stress, immune regulation, gut microbial metabolism, and epigenetic remodeling. These interconnected pathways dynamically interact with intrinsic tumor characteristics, host physiology, and environmental exposures, thereby contributing to the remarkable heterogeneity observed in breast cancer susceptibility and clinical outcomes. Increasing recognition of these multidimensional interactions has prompted a conceptual shift from studying individual nutrients toward understanding integrated metabolic and endocrine ecosystems that collectively determine cancer risk and therapeutic responsiveness.

The concept of using nutritional biomarkers as objective indicators of dietary exposure, metabolic status, and their association with tumor biology has gained considerable traction in recent years (5). Unlike self-reported dietary assessments, which are subject to recall bias and measurement error, nutritional biomarkers provide a more accurate and quantitative measure of nutrient intake, absorption, and utilization. These biomarkers can be categorized into various types (6), including recovery biomarkers that reflect short-term nutrient intake (7), concentration biomarkers that indicate body stores or tissue levels (8), replacement biomarkers that represent functional biological effects (9), and predictive biomarkers that forecast disease risk or progression (10). However, the field currently faces several critical gaps: existing reviews typically examine individual biomarker classes in isolation, without establishing a unifying classification framework that bridges functional biomarker categories with molecular subtype-specific prognostic relevance; pre-analytical confounding factors that substantially influence biomarker measurement—including sampling conditions, fasting status, specimen handling, and racial disparities—remain systematically underdiscussed, impeding standardization and clinical translation; the predictive dimensions of different prognostic model performance metrics (C-index, time-dependent AUC, calibration, DCA) are rarely interpreted in clinically meaningful terms; and the evidentiary basis for precision nutrition strategies remains heavily skewed toward preclinical and observational studies, with insufficient acknowledgment of how this constrains near-term clinical applicability (**Figure 1**).

**Figure 1:**
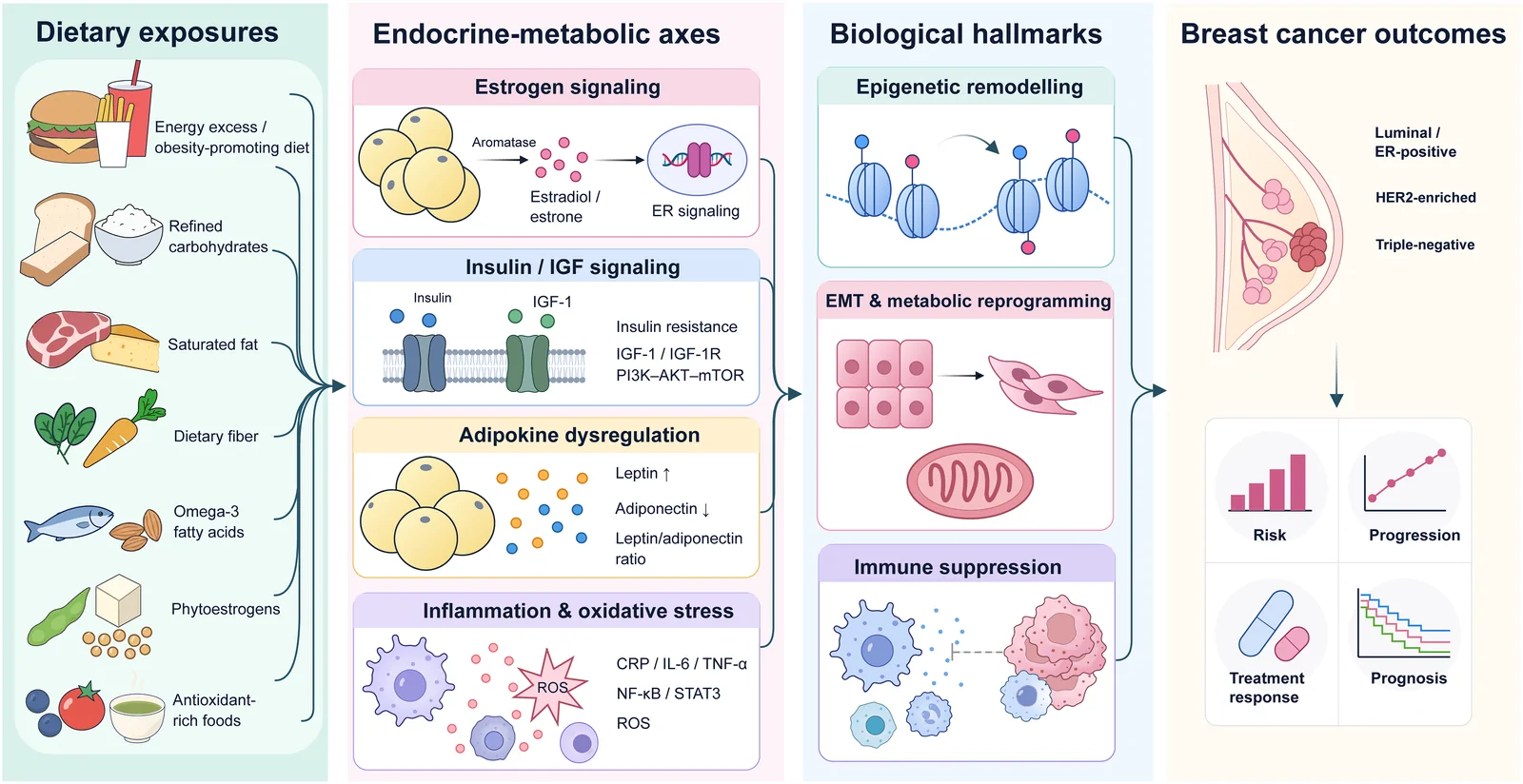
Schematic linking dietary exposures to breast cancer via four interconnected endocrine-metabolic axes (estrogen, insulin/IGF, adipokine, and inflammatory/oxidative stress), driving downstream hallmarks (epigenetics, EMT, immune suppression) and dictating subtype-specific prognosis.

Importantly, the value of nutritional biomarkers extends far beyond serving as passive indicators of dietary exposure. Many biomarkers participate directly in cellular signaling networks governing proliferation, apoptosis, DNA repair, mitochondrial function, oxidative metabolism, and immune regulation. Consequently, they may simultaneously function as exposure biomarkers, mechanistic biomarkers, prognostic biomarkers, and potentially predictive biomarkers for therapeutic response. Advances in high-throughput metabolomics, lipidomics, proteomics, and systems biology have further accelerated the discovery of composite biomarker signatures capable of capturing complex metabolic states that cannot be adequately represented by single nutritional measurements. Such multidimensional biomarker panels provide unprecedented opportunities to integrate nutritional status with molecular characteristics of tumors, thereby facilitating more accurate biological stratification and individualized clinical decision-making.

The endocrine system plays a central role in mediating the effects of nutrition on breast cancer development and progression. Key mechanisms include estrogen signaling, insulin resistance, adipokine regulation, and inflammatory pathways (11). Obesity, which represents a state of nutritional excess and metabolic dysregulation, exemplifies this connection, with multiple studies demonstrating its association with increased breast cancer risk and worse prognosis, particularly in postmenopausal women (12). The mechanisms underlying this association involve increased insulin-like growth factor (IGF)-I bioavailability, oxidative stress, altered leptin-to-adiponectin ratio, and increased inflammatory cytokines (13) that create a systemic and local hyperestrogenic milieu through the induction of aromatase activity.

Beyond these classical endocrine pathways, accumulating evidence indicates that endocrine regulation should be viewed as an integrated biological network rather than a collection of isolated hormonal axes. Crosstalk among estrogen receptors, insulin/IGF signaling, adipokines, inflammatory mediators, circadian hormones, and gut microbiota-derived metabolites generates highly interconnected signaling circuits capable of influencing cellular plasticity, metabolic reprogramming, epithelial-mesenchymal transition, immune escape, and therapeutic sensitivity. Nutritional factors may therefore exert synergistic or antagonistic effects across multiple endocrine pathways simultaneously, amplifying or attenuating breast cancer progression depending on host metabolic status and tumor molecular subtype. This systems-level perspective helps explain why apparently inconsistent epidemiological findings often emerge when individual nutrients are investigated without considering broader metabolic and endocrine contexts.

Meanwhile, the rapid development of precision medicine has transformed expectations regarding biomarker-guided prevention and treatment. Conventional prognostic factors—including tumor stage, histological grade, and receptor status—provide only limited insight into host metabolic conditions that substantially influence disease evolution and treatment efficacy. Integrating nutritional biomarkers with endocrine characteristics, metabolic phenotypes, and molecular tumor profiles offers the possibility of constructing more comprehensive prediction models capable of identifying individuals at elevated risk, refining prognostic assessment, predicting treatment response, and monitoring disease progression over time. Such integrative strategies align closely with the emerging concept of precision nutrition, in which dietary recommendations are tailored according to an individual’s biological characteristics rather than generalized population guidelines.

This review aims to synthesize current evidence on nutritional biomarkers and their interactions with endocrine-related mechanisms in breast cancer, with a specific focus on risk assessment and prognosis prediction. We will explore the biological foundations of these interactions, examine specific biomarkers with clinical utility, discuss the integration of biomarkers into prediction models, and highlight future directions for research and clinical application. By systematically connecting functional biomarker classifications with subtype-specific metabolic signatures and prognostic model performance, this review provides a comprehensive framework for understanding how nutritional biomarkers can advance precision medicine in breast cancer (**Figure 2**).

**Figure 2:**
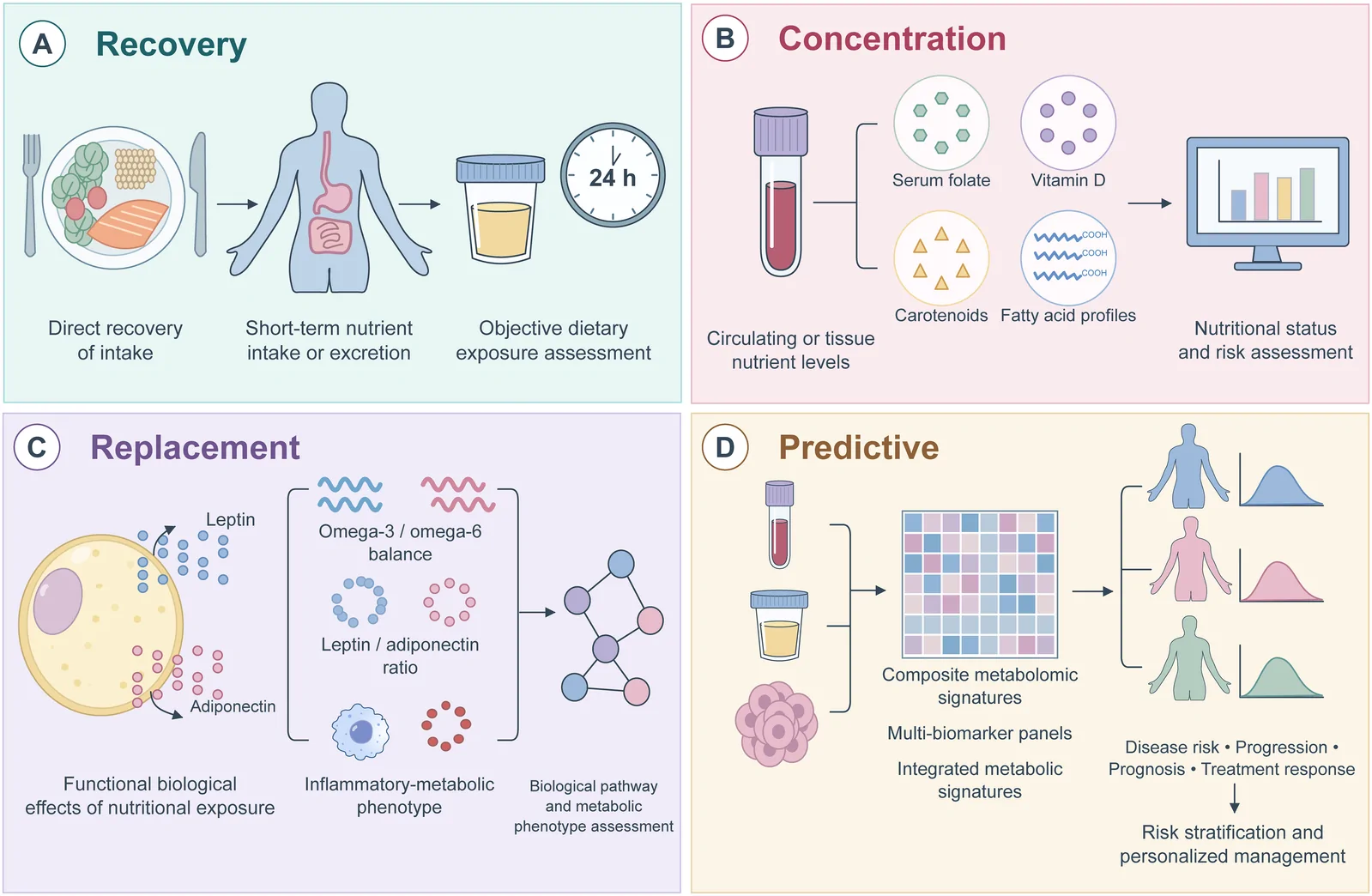
Functional classification of nutritional biomarkers (recovery, concentration, replacement, predictive), detailing their distinct biological definitions, representative examples, and specific translational applications in breast cancer risk stratification.

## 2 Biological foundations of diet-endocrine interactions in breast cancer

The four core endocrine-metabolic axes linking nutrition to breast cancer—obesity/adipokine dysregulation, insulin/IGF signaling, estrogen metabolism, and inflammatory-oxidative stress—operate as an integrated network rather than independent pathways. For each axis, we focus specifically on: (i) which dietary factors modulate the axis, (ii) the key circulating biomarkers that reflect such regulation, and (iii) the downstream biological consequences for breast cancer biology.

### 2.1 Obesity, metabolic dysregulation, and breast cancer

Obesity, defined by the excessive accumulation of body fat, is widely recognized as a multifaceted condition encompassing chronic low-grade inflammation and metabolic dysregulation (14). Epidemiological studies have consistently demonstrated a robust correlation between obesity and an escalated risk of breast cancer (15), particularly in the postmenopausal demographic (16). This association is further compounded by poorer prognoses and diminished survival rates among obese women diagnosed with breast cancer when juxtaposed with their normal-weight counterparts. The etiological mechanisms facilitating this link are intricate and multi-tiered, involving pathways such as insulin resistance, perturbations in adipokine secretion, persistent inflammation, and hormonal imbalances.

Key dietary regulators of this axis include excessive caloric intake, high saturated fat consumption, low dietary fiber, and imbalanced omega-6/omega-3 fatty acid ratios. These dietary patterns promote adipose tissue expansion, adipocyte hypertrophy, and subsequent endocrine dysfunction. The primary circulating biomarkers reflecting obesity-associated metabolic dysregulation include leptin, adiponectin, the leptin-to-adiponectin ratio, resistin, and free fatty acid profiles.

Increasing evidence suggests that obesity should no longer be regarded simply as excessive fat accumulation but rather as a systemic metabolic disorder characterized by profound endocrine, immune, and metabolic remodeling. Adipose tissue functions as a dynamic endocrine organ that actively communicates with distant organs through hormones, cytokines, lipid mediators, extracellular vesicles, and metabolites. Consequently, obesity induces widespread alterations in systemic metabolic homeostasis that influence virtually every hallmark of cancer, including sustained proliferative signaling, resistance to cell death, angiogenesis, immune evasion, metabolic adaptation, and metastatic dissemination. Rather than acting independently, these biological alterations establish an integrated metabolic environment that facilitates breast tumor initiation and accelerates disease progression.

The relationship between obesity and breast cancer is highly context-dependent and varies according to menopausal status, molecular subtype, body fat distribution, and metabolic health. While obesity generally increases the incidence of hormone receptor-positive breast cancer after menopause, metabolically unhealthy obesity is increasingly recognized as a particularly aggressive phenotype regardless of body mass index. Conversely, certain individuals with normal body weight may exhibit significant metabolic abnormalities, including visceral adiposity, insulin resistance, and chronic inflammation, suggesting that metabolic dysfunction may represent a stronger determinant of breast cancer biology than adiposity alone. These observations have shifted research emphasis from anthropometric measurements toward functional metabolic biomarkers capable of more accurately reflecting cancer-associated metabolic risk.

Adipose tissue in postmenopausal women acts as the predominant site for estrogen synthesis, facilitated through aromatase-mediated conversion of androgens to estrogens (17). The adipose tissue hypertrophy characteristic of obesity leads to an upregulation of systemic estrogen levels, which in turn (18), fuels the growth and proliferation of hormone receptor-positive breast cancer cells. Furthermore, the obese state is often accompanied by hyperleptinemia and a hypoadiponectinemia (19), establishing an unfavorable adipokine milieu conducive to tumorigenesis and tumor progression. Leptin, with its mitogenic, angiogenic, and pro-inflammatory effects, is instrumental in stimulating breast cancer cell proliferation and invasion (20). Conversely, adiponectin’s insulin-sensitizing and anti-proliferative properties are thwarted in obesity, with lower levels correlating with an enhanced cancer risk (21).

The leptin-to-adiponectin ratio has emerged as a particularly informative functional biomarker, as it captures the net balance between pro-tumorigenic and anti-tumorigenic adipose tissue signals. Elevated ratios are consistently associated with increased breast cancer risk and poorer prognosis, particularly in postmenopausal women with hormone receptor-positive disease (18, 20).

Beyond alterations in circulating hormone concentrations, obesity profoundly remodels intracellular signaling networks within breast epithelial cells. Hyperleptinemia promotes activation of multiple oncogenic pathways that regulate cellular proliferation, migration, epithelial-mesenchymal transition, and cancer stem cell maintenance, whereas reduced adiponectin weakens AMP-activated protein kinase (AMPK)-mediated metabolic regulation and diminishes protective anti-inflammatory signaling. The imbalance between leptin and adiponectin therefore represents more than a simple endocrine alteration; it reflects a comprehensive shift toward anabolic metabolism, enhanced biosynthesis, and increased cellular survival. Consequently, the leptin-to-adiponectin ratio has emerged as a functional indicator of obesity-associated endocrine dysfunction and provides a mechanistic bridge linking nutritional status with breast cancer progression.

Obesity also exerts profound effects on systemic lipid metabolism. Excess circulating free fatty acids released from hypertrophic adipocytes contribute to hepatic lipid accumulation, ectopic fat deposition, mitochondrial dysfunction, and oxidative stress. Tumor cells exhibit remarkable metabolic plasticity and are capable of utilizing these circulating lipids as alternative energy substrates through enhanced fatty acid uptake and β-oxidation. Such metabolic flexibility enables malignant cells to adapt to nutrient fluctuations within the tumor microenvironment while simultaneously supporting rapid membrane biosynthesis, ATP production, and redox homeostasis. Consequently, obesity provides not only endocrine stimulation but also abundant metabolic substrates that facilitate sustained tumor growth.

The significance of peritumoral adipose tissue in breast cancer development is now more clearly delineated through recent investigative findings (22, 23). In the proximity of tumor cells, adipocytes undergo metabolic reprogramming, marked by augmented lipolysis and the consequent discharge of free fatty acids. These fatty acids serve as an alternate energy supply for cancer cells, fostering a metabolic symbiosis that perpetuates tumor growth and invasiveness (22, 24). Moreover, adipose tissue hypoxia, a consequence of tissue expansion, triggers the transcription of hypoxia-inducible factors (HIFs) (25). The resultant overexpression of HIFs engenders the activation of pro-tumorigenic pathways, notably those involved in the epithelial-mesenchymal transition (EMT) and angiogenesis (26), further intensifying the carcinogenic landscape. Collectively, these discoveries delineate adipose tissue as more than a mere passive caloric reservoir; rather, it is an active endocrine organ exerting a profound impact on the biology of breast cancer (27, 28).

Recent investigations further indicate that cancer-associated adipocytes undergo phenotypic dedifferentiation after interacting with neighboring breast cancer cells. These adipocytes display reduced lipid content, increased secretion of inflammatory cytokines, extracellular matrix-remodeling enzymes, and chemokines, thereby generating a permissive microenvironment that enhances tumor invasion and metastatic dissemination. Simultaneously, reciprocal communication between adipocytes and cancer cells establishes positive feedback loops that reinforce metabolic coupling. Tumor-derived factors stimulate adipocyte lipolysis, whereas adipocyte-derived metabolites fuel oxidative metabolism within malignant cells, ultimately forming a self-sustaining metabolic ecosystem that promotes tumor aggressiveness.

The influence of obesity extends beyond adipocytes themselves and profoundly reshapes the immune landscape of adipose tissue. Obesity is associated with increased infiltration of macrophages, neutrophils, myeloid-derived suppressor cells, and dysfunctional lymphocyte populations, leading to persistent low-grade inflammation. These immune cells release abundant inflammatory mediators that amplify aromatase activity, promote extracellular matrix remodeling, and stimulate angiogenesis. At the same time, chronic inflammatory signaling contributes to T-cell exhaustion and impaired antitumor immunity, allowing transformed cells to evade immune surveillance during early tumorigenesis. This reciprocal interaction between metabolic dysfunction and immune dysregulation forms the basis of the emerging concept of immunometabolism in breast cancer.

Metabolic dysregulation associated with obesity also contributes to substantial alterations in mitochondrial function and cellular bioenergetics. Increased oxidative stress, impaired mitochondrial quality control, excessive reactive oxygen species production, and disrupted nutrient sensing collectively induce genomic instability and epigenetic remodeling. These metabolic abnormalities facilitate the acquisition of malignant phenotypes while simultaneously promoting resistance to apoptosis and adaptation to hypoxic conditions. Importantly, these metabolic alterations frequently precede clinically detectable disease, highlighting their potential utility as early biomarkers for breast cancer risk assessment and prevention.

Dietary interventions targeting this axis—including caloric restriction, omega-3 fatty acid supplementation, and Mediterranean dietary patterns—have been shown to improve leptin/adiponectin balance, reduce systemic inflammation, and decrease aromatase activity in adipose tissue (18). These effects provide a mechanistic rationale for obesity-focused nutritional strategies in breast cancer prevention and management.

Collectively, obesity represents a complex systemic disorder characterized by coordinated disturbances in endocrine signaling, lipid metabolism, inflammatory activation, immune regulation, and cellular energetics rather than merely excessive body weight. These interconnected biological processes establish a metabolically favorable environment that supports breast cancer initiation, progression, and therapeutic resistance. Consequently, obesity-associated metabolic dysfunction should be viewed as a dynamic and potentially modifiable component of breast cancer biology. A deeper understanding of these integrated mechanisms provides a strong biological rationale for biomarker-guided metabolic risk stratification and supports the development of precision nutritional interventions targeting multiple endocrine pathways simultaneously, thereby laying the mechanistic foundation for the insulin/IGF signaling, estrogen metabolism, and inflammatory pathways discussed in the following sections.

### 2.2 Insulin resistance and IGF signaling

The intricate relationship between insulin and insulin-like growth factor (IGF) systems is pivotal in connecting metabolic health to the risk and progression of breast cancer (29). Diets high in refined carbohydrates, added sugars, and saturated fats are the primary dietary drivers of insulin resistance. The key biomarkers reflecting dysregulation of this axis include fasting insulin, C-peptide, HOMA-IR, circulating IGF-1, IGF-binding proteins (IGFBPs), and the triglyceride-glucose (TyG) index (4, 7, 19). Obesity and consumption of diets high in refined carbohydrates and saturated fats are known to precipitate insulin resistance, a metabolic derangement characterized by diminished tissue sensitivity to insulin (30). This resistance spurs a compensatory hyperinsulinemic response, which in turn has been noted to be independently associated with an escalated risk of breast cancer and an increased likelihood of mortality.

Insulin resistance has emerged as one of the central metabolic abnormalities linking nutritional imbalance with carcinogenesis. Rather than representing an isolated endocrine disorder, insulin resistance reflects a systemic metabolic state characterized by impaired glucose utilization, compensatory hyperinsulinemia, dysregulated lipid metabolism, chronic inflammation, and mitochondrial dysfunction. These interconnected abnormalities collectively generate a biochemical environment that favors malignant transformation and sustained tumor growth. Consequently, increasing attention has shifted from evaluating insulin concentrations alone toward understanding the broader metabolic network in which insulin signaling interacts dynamically with endocrine, inflammatory, and nutritional pathways.

Hyperinsulinemia influences breast cancer development through both direct and indirect mechanisms. On one hand, elevated circulating insulin exerts mitogenic effects by acting directly on mammary epithelial cells. On the other hand, insulin profoundly alters the endocrine milieu by modulating hepatic metabolism, adipose tissue function, and sex hormone bioavailability. These systemic metabolic alterations establish a permissive environment that enhances cellular proliferation while simultaneously suppressing multiple physiological mechanisms responsible for maintaining metabolic homeostasis.

Hyperinsulinemia, through its direct influence on cancer cell proliferation, acts by engaging the insulin receptor (IR) and instigating its downstream signaling pathways (31), namely the RAS/MAPK and PI3K/AKT pathways (29, 31). These cascades are integral in regulating vital cellular processes such as cell growth, proliferation, metabolism, and survival (32). Moreover, insulin’s impact on estrogen signaling through modulation of aromatase activity and sex hormone-binding globulin (SHBG) production can lead to an increase in bioavailable estrogen levels (33), which is a significant consideration due to the estrogen receptor’s role in breast cancer development.

Activation of PI3K/AKT signaling represents one of the principal molecular events linking insulin resistance with breast tumorigenesis. Beyond promoting proliferation and inhibiting apoptosis, this pathway orchestrates profound metabolic reprogramming by stimulating glucose uptake, glycolysis, lipid biosynthesis, protein translation, and nucleotide synthesis (30, 32). Simultaneously, activation of mTOR signaling enhances anabolic metabolism, allowing tumor cells to efficiently utilize nutrients for rapid biomass accumulation. In parallel, the RAS/MAPK pathway primarily regulates cellular proliferation (15), differentiation, and survival, while substantial crosstalk between these signaling networks creates robust compensatory mechanisms that maintain malignant growth even under therapeutic pressure (33). The convergence of these pathways highlights how metabolic signaling extends far beyond glucose regulation to influence nearly every aspect of tumor biology.

An additional consequence of chronic hyperinsulinemia is the disruption of hepatic endocrine regulation. Elevated insulin suppresses hepatic production of sex hormone-binding globulin, thereby increasing circulating free estrogen concentrations and amplifying estrogen receptor signaling. Simultaneously, insulin stimulates ovarian androgen production in premenopausal women while enhancing peripheral aromatase activity within adipose tissue, further increasing estrogen biosynthesis. These endocrine alterations establish a biological bridge between metabolic dysfunction and hormone-dependent breast carcinogenesis, illustrating why obesity, insulin resistance, and estrogen signaling frequently coexist and synergistically promote disease progression.

The IGF system, and particularly IGF-1 (34), exhibits structural homology with insulin, allowing it to activate analogous signaling pathways. These pathways bestow potent mitogenic and anti-apoptotic effects on breast epithelial cells, thus contributing to neoplastic growth (35, 36). The intersection of insulin/IGF signaling with estrogen receptor (ER) pathways creates a milieu that is propitious for tumorigenesis (37).

The IGF axis constitutes a highly coordinated endocrine network comprising IGF ligands, IGF-binding proteins (IGFBPs), membrane receptors, and intracellular adaptor molecules (38). Rather than functioning independently, this network tightly regulates cell-cycle progression, differentiation, tissue repair, and metabolic adaptation under physiological conditions. During breast carcinogenesis, however, dysregulation of the IGF system results in persistent activation of growth-promoting signaling cascades. Increased IGF bioavailability, altered expression of IGFBPs, and enhanced receptor activation collectively stimulate continuous proliferative signaling while reducing apoptotic susceptibility (28, 30). Consequently, tumor cells acquire greater metabolic flexibility and increased capacity to survive under hypoxic or nutrient-limited conditions.

Emerging evidence further indicates extensive reciprocal interactions between insulin/IGF signaling and multiple oncogenic pathways. Crosstalk with estrogen receptor signaling enhances transcription of proliferation-associated genes, whereas interactions with inflammatory mediators amplify NF-κB-dependent cytokine production and chronic inflammatory responses (26). Moreover, activation of insulin/IGF signaling influences epithelial-mesenchymal transition, angiogenesis, extracellular matrix remodeling, and cancer stem cell maintenance through coordinated regulation of multiple downstream effectors. These interconnected signaling networks illustrate that insulin resistance functions as a central metabolic hub rather than an isolated endocrine abnormality.

Recent research (38) has shed light on the nuanced interplay between insulin resistance and specific molecular subtypes of breast cancer. Triple-negative breast cancer (TNBC) (39), which constitutes around 15–20% of all breast cancers and is defined by the absence of ER, progesterone receptor (PR), and HER2 expression, seems to be especially dependent on IGF signaling for its proliferation and survival (40). This subtype, known for its basal-like gene expression patterns, demonstrates a heightened sensitivity to IGF-1 receptor inhibition in preclinical assays (41). In addition, obesity-associated hyperinsulinemia may contribute to resistance to therapy in hormone receptor-positive breast cancers by supplying alternative growth signals that subvert endocrine therapy’s ER blockade (42). These subtype-specific differences have important implications for biomarker selection: insulin/IGF-axis biomarkers may carry greater prognostic weight in TNBC, whereas their predictive value for endocrine therapy response is most relevant in ER-positive disease.

Growing evidence suggests that metabolic heterogeneity among breast cancer subtypes may partially explain differences in prognosis and therapeutic response. Hormone receptor-positive tumors frequently exhibit strong interactions between insulin signaling and estrogen-dependent transcriptional programs, whereas triple-negative tumors rely more heavily on metabolic plasticity to satisfy their elevated energetic demands (43). In HER2-positive disease, insulin/IGF signaling may cooperate with receptor tyrosine kinase pathways to enhance proliferative capacity and reduce therapeutic sensitivity (44). These observations emphasize that metabolic abnormalities do not uniformly influence all breast cancers but instead interact with subtype-specific molecular programs to shape tumor behavior.

Beyond influencing tumor initiation, insulin resistance increasingly appears to contribute to therapeutic resistance. Persistent activation of PI3K/AKT/mTOR signaling provides alternative survival pathways that diminish the effectiveness of endocrine therapy, targeted therapy, and even chemotherapy (45, 46). Furthermore, hyperinsulinemia promotes metabolic adaptation by increasing nutrient uptake, mitochondrial plasticity, and antioxidant capacity, enabling tumor cells to better withstand therapeutic stress. These adaptive mechanisms may explain why patients with obesity or diabetes often experience poorer treatment responses and less favorable clinical outcomes.

From a translational perspective, biomarkers reflecting insulin resistance—including fasting insulin, C-peptide, HOMA-IR, circulating IGF-1, and composite metabolic signatures (26, 35, 36, 39) —have attracted considerable interest for risk stratification and prognostic prediction. Although no single biomarker has yet demonstrated sufficient specificity for routine clinical implementation, integrating insulin-related biomarkers with adipokines, inflammatory mediators, and metabolomic profiles may substantially improve individualized prediction models (47). Such multidimensional metabolic assessment aligns with the emerging paradigm of precision nutrition and precision oncology, in which metabolic phenotyping complements traditional clinicopathological variables to guide prevention, prognosis, and therapeutic decision-making (48).

Collectively, insulin resistance and IGF signaling represent fundamental biological mechanisms linking nutritional imbalance with breast cancer development. Their influence extends far beyond glucose metabolism to encompass endocrine regulation, metabolic reprogramming, inflammatory activation, immune modulation, and therapeutic responsiveness (49). Understanding these integrated signaling networks provides a critical mechanistic foundation for interpreting nutritional biomarkers and supports the development of metabolism-oriented preventive and therapeutic strategies (50). Furthermore, the intimate interaction between insulin/IGF pathways and estrogen metabolism naturally leads to the next section, which discusses the central role of estrogen signaling and its nutritional regulation in breast cancer pathogenesis.

### 2.3 Estrogen signaling and metabolism

Estrogen, a hormone with pivotal influence, is intricately involved in the pathogenesis of breast cancer, predominantly amongst the estrogen receptor (ER)-positive subset (43). Multiple dietary factors modulate estrogen metabolism, including phytoestrogens (isoflavones, lignans), dietary fiber, cruciferous vegetable-derived indoles, dietary fat composition, and alcohol (36, 39, 44). The primary biomarkers reflecting estrogen axis regulation include circulating estradiol, estrone, SHBG, 2-hydroxyestrone/16α-hydroxyestrone ratio, and urinary estrogen metabolite profiles (44, 45). The intricate relationship between nutritional habits and estrogen metabolism serves as a cornerstone linking dietary factors to breast cancer susceptibility (44). This nexus suggests that dietary choices have the capacity to alter systemic estrogen levels through a variety of mechanisms such as the modulation of the key enzyme aromatase, which catalyzes the conversion of androgens to estrogens (45), adjustments to the enterohepatic circulation, a sequence involving the reabsorption and recycling of estrogens in the liver, and impact on the metabolic enzymes and pathways that process estrogens.

Estrogen signaling is increasingly recognized as a dynamic endocrine network rather than a single hormonal pathway. In addition to regulating mammary gland development under physiological conditions, estrogen profoundly influences cell proliferation, apoptosis, differentiation, DNA repair, and metabolic homeostasis (46). Nutritional status can modify these biological processes through alterations in hormone synthesis, receptor activation, and downstream transcriptional regulation, thereby affecting both breast cancer initiation and progression (47, 48). Consequently, the interaction between diet and estrogen metabolism represents a critical interface through which environmental exposures influence breast cancer risk.

Phytoestrogens, plant-originated molecules possessing structural similarities with endogenous estrogens, exemplify the complex interplay between diet and endocrine function in breast carcinogenesis (46). These compounds, encompassing isoflavones and lignans, exhibit the capacity to elicit both estrogenic and anti-estrogenic effects. Such effects are contingent upon their concentration, the prevailing endocrine milieu, and the specific characteristics of individuals (47). Empirical evidence from epidemiological studies supports the notion that diets rich in phytoestrogens may confer a protective advantage against breast cancer, an association that is particularly pronounced in Asian populations (48). However, a congruent consensus is yet to emerge, as study outcomes have exhibited variability.

This variability may partially reflect differences in dietary patterns, gut microbial metabolism, menopausal status, and endogenous estrogen concentrations among populations. Rather than functioning as direct estrogen substitutes, phytoestrogens often act as selective modulators of estrogen receptor activity, producing tissue-specific biological responses under different endocrine environments (49). Such context-dependent effects may explain the inconsistent associations reported across epidemiological studies and highlight the complexity of evaluating dietary components in hormone-dependent cancers (50–52).

Dietary constituents, such as fiber content, also bear significance in modulating estrogen metabolism. They do so by influencing the activity of β-glucuronidase [53, 54. 55], a key enzyme in estrogen reprocessing, and fecal excretion, thereby exerting control over the levels of circulating estrogens. The interplay between nutrition and endocrine function is thus not only complex but also holds potential interventional value in the management of breast cancer.

The gut microbiome is increasingly recognized as a critical regulator of estrogen metabolism through its control over the “estrobolome” (53, 54)—the collection of microbial genes responsible for estrogen metabolism. Dietary fiber, prebiotics, and polyphenols shape gut microbial composition and β-glucuronidase activity, thereby modulating enterohepatic estrogen recirculation (46, 54, 55). This diet–microbiome–estrogen axis represents an important but understudied pathway through which nutritional factors influence breast cancer risk.

Beyond regulating estrogen availability, dietary factors may also influence estrogen receptor signaling indirectly through alterations in systemic metabolism and inflammatory status (55). Obesity-associated increases in aromatase activity, together with insulin resistance and adipokine dysregulation, further enhance estrogen biosynthesis within adipose tissue, creating a microenvironment favorable for hormone-dependent tumor growth (56). These observations reinforce the concept that estrogen metabolism is closely integrated with other endocrine pathways rather than functioning independently.

New horizons in research are elucidating the significance of estrogen receptor (ESR1) mutations in the resistance exhibited to endocrine therapies (50, 51). It is estimated that a considerable proportion of ER-positive metastatic breast cancers that demonstrate resistance to endocrine therapies harbor these ESR1 mutations. These mutations are instrumental in the constitutive activation of the estrogen receptor signaling pathway, thereby circumventing the requirement for physiological ligand binding (52). Interestingly, these mutations tend to emerge under the selective pressure exerted by ongoing endocrine therapies, underscoring their pivotal role in therapeutic resistance (57). Nonetheless, this presents an avenue for intervention. Dietary components, such as indole-3-carbinol (58) derived from cruciferous vegetables, have shown promise in modulating the function of mutant estrogen receptors in preclinical settings (59). This suggests that targeted nutritional interventions may synergize with conventional therapeutic strategies.

The emergence of ESR1 mutations further illustrates the dynamic interaction between tumor evolution and endocrine signaling. As breast cancers acquire resistance during treatment, metabolic and nutritional factors may continue to influence tumor behavior by modifying receptor activity or downstream signaling pathways. Although clinical evidence remains limited, integrating endocrine biomarkers with nutritional status may improve patient stratification and facilitate more individualized therapeutic strategies.

Moreover, the gut microbiome is increasingly recognized as a regulator of estrogen metabolism. It does so through its control over the “estrobolome” (53, 54) encompassing the microbial genes responsible for the metabolism of estrogens. This revelation points towards the potential of dietary manipulation of the gut microbiota to become a novel strategy for modulating estrogenic levels and activity, thus offering a fertile ground for future preventive and therapeutic applications in breast cancer management.

Accumulating evidence suggests that the gut microbiota serves as an important mediator linking diet, endocrine homeostasis, and breast cancer. Alterations in microbial composition may influence estrogen recycling, inflammatory responses, and the production of bioactive metabolites, thereby modifying systemic hormone exposure. As microbiome research continues to evolve, integrating nutritional biomarkers with endocrine and microbial signatures may provide a more comprehensive understanding of breast cancer biology and support the development of precision nutrition strategies for disease prevention and prognosis (55, 56).

### 2.4 Inflammation and oxidative stress

The inflammatory response is a complex biological process that plays a crucial role in the development and progression of cancer, particularly within the context of obesity (55). Dietary patterns are the primary modulators of this axis: the Mediterranean diet (rich in fruits, vegetables, whole grains, and omega-3 fatty acids) exerts anti-inflammatory effects, whereas Western-type diets (high in saturated fats, refined carbohydrates, and processed meats) promote chronic low-grade inflammation (60). Key biomarkers reflecting inflammatory-oxidative status include CRP, IL-6, TNF-α, NF-κB activity markers, and oxidative stress markers such as 8-OHdG and malondialdehyde (61, 62). Obesity-associated chronic inflammation is characterized by an increased infiltration of adipose tissue with immune cells (56), primarily macrophages, and the overproduction of pro-inflammatory cytokines (60), such as tumor necrosis factor-alpha (TNF-α) and interleukin-6 (IL-6). These cytokines are potent mediators that activate transcription factors like nuclear factor-kappa B (NF-κB) (61) and signal transducer and activator of transcription 3 (STAT3) (62, 63), thereby regulating the expression of genes involved in critical cancer-promoting processes such as cell proliferation, apoptosis evasion, angiogenesis, and metastasis.

Chronic low-grade inflammation is now recognized as a hallmark linking metabolic dysfunction with breast cancer development. Unlike acute inflammation, which is generally self-limited and protective, persistent inflammatory activation creates a systemic microenvironment that favors genomic instability, aberrant tissue remodeling, and sustained proliferative signaling (45, 55). Continuous activation of NF-κB and STAT3 not only promotes tumor cell survival but also establishes positive feedback loops between inflammatory cells, adipocytes, and breast cancer cells, thereby amplifying cytokine production and maintaining chronic inflammatory signaling throughout tumor progression (64, 65).

A pivotal link between nutritional status and cancer development is the modulation of inflammation through dietary patterns (64). The Mediterranean diet, known for its rich content of fruits, vegetables, whole grains, and omega-3 fatty acids (65, 66), has been associated with lower inflammation levels and a reduced risk of breast cancer. Its anti-inflammatory effects are often attributed to the presence of omega-3 fatty acids, notably eicosapentaenoic acid (EPA) and docosahexaenoic acid (DHA) (65), which competitively inhibit arachidonic acid metabolism by cyclooxygenase and lipoxygenase enzymes, consequently leading to the generation of less inflammatory eicosanoids (67). Furthermore, dietary antioxidants present in fruits and vegetables are known to combat oxidative stress, thereby reducing DNA damage and potentially inhibiting cancer initiation and progression (68, 69) (**Figure 3**).

**Figure 3:**
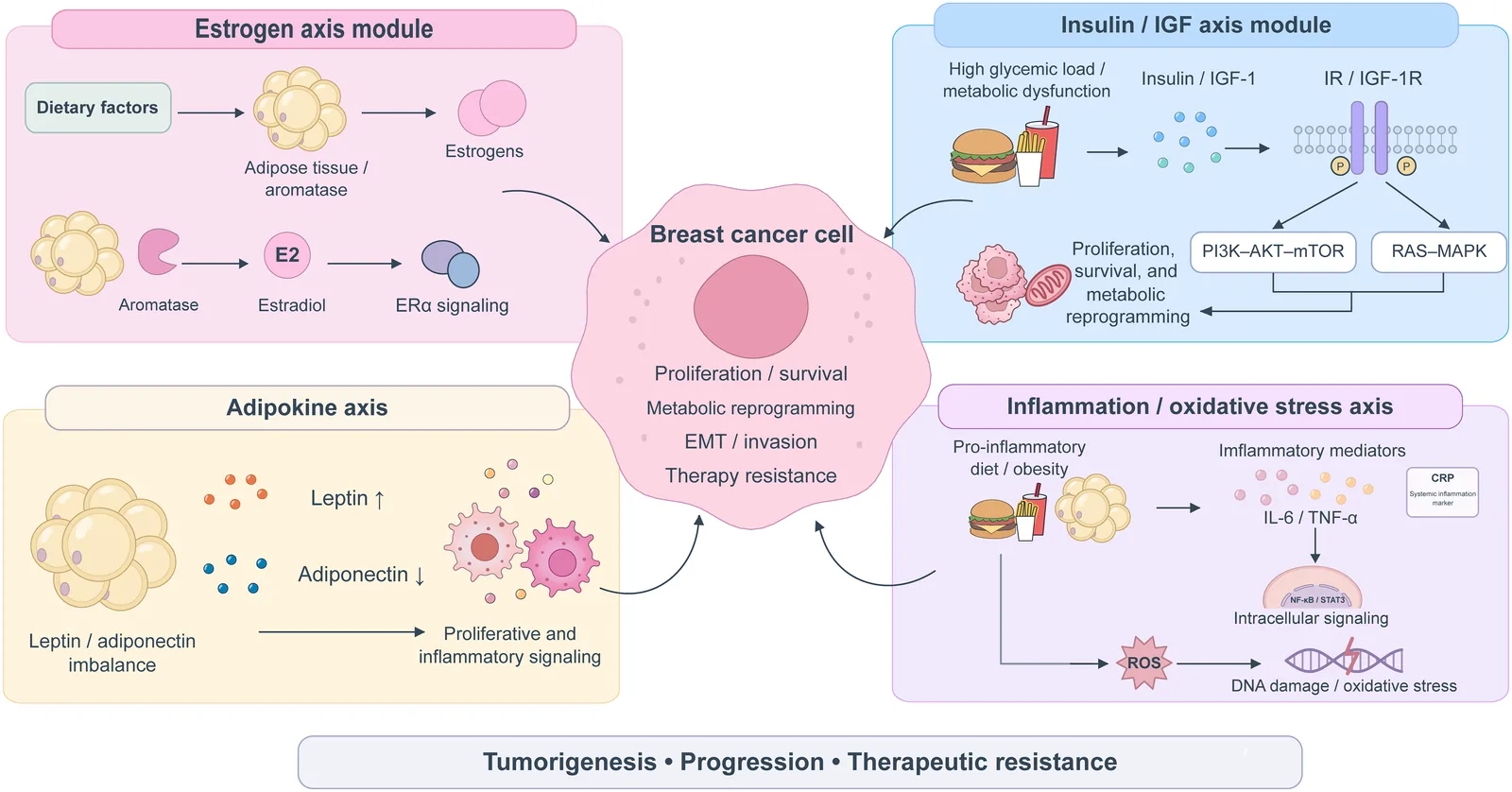
Core molecular crosstalk among the four signaling axes. Diet modulates aromatase (Estrogen), IGF-1R/PI3K (Insulin), Leptin/Adiponectin (Adipokine), and NF-κB/STAT3 (Inflammation), converging to promote tumorigenesis and therapeutic resistance.

Beyond individual nutrients, overall dietary patterns substantially influence inflammatory homeostasis by regulating immune cell activity, redox balance, and metabolic function. Diets rich in plant-derived antioxidants, dietary fiber, and unsaturated fatty acids contribute to maintaining redox equilibrium and suppressing excessive inflammatory signaling, whereas long-term consumption of energy-dense diets promotes oxidative stress and metabolic dysfunction (70). These findings suggest that nutritional interventions may modify breast cancer risk not only through endocrine regulation but also by reshaping systemic inflammatory and oxidative environments.

Western-type diets, in contrast, are high in saturated fats, refined carbohydrates, and processed meats, and are known to induce inflammation and oxidative stress. These diets contribute to an environment that promotes the occurrence and progression of cancer by sustaining an inflammatory state and increasing the production of reactive oxygen species (ROS) (70, 71), which can cause DNA damage and genomic instability.

Persistent oxidative stress further accelerates breast carcinogenesis through multiple biological mechanisms. Excessive ROS production damages DNA, proteins, and membrane lipids, while simultaneously altering mitochondrial function and intracellular signaling pathways (53, 70). Moderate levels of ROS may also function as signaling molecules that stimulate proliferation, angiogenesis, and metabolic adaptation, illustrating the dual role of oxidative stress in tumor biology (71). Consequently, maintaining redox homeostasis represents an important mechanism through which nutritional factors may influence both cancer initiation and disease progression.

The intricate interplay between nutrition-induced inflammation and the tumor immune microenvironment has been the subject of recent scrutiny. In breast cancer, obesity-associated inflammation is further characterized by alterations in immune cell distribution, including the increased presence of tumor-associated macrophages (TAMs) (72) and regulatory T cells (Tregs) (73), which foster an immunosuppressive tumor microenvironment conducive to cancer progression. Notably, a recent study identified CRISP3 as a biomarker in breast cancer that is related to hypoxia, epithelial-mesenchymal transition (EMT) (73, 74), and immune responses. High expression levels of CRISP3 (75, 76) are associated with increased immune cell infiltration but lead to a decreased response to immunotherapy, suggesting a complex relationship between nutritional inflammation and the efficacy of cancer immunotherapies. This illustrates the need to consider the broader impact of nutritional interventions on the tumor-immune interface.

These observations emphasize that inflammatory biomarkers may possess clinical value beyond reflecting systemic inflammation. They provide important insights into the dynamic interactions among metabolism, immunity, and tumor biology, and may contribute to identifying patients with distinct immunometabolic phenotypes. Such information may ultimately improve risk stratification and facilitate the development of personalized nutritional or immunomodulatory strategies.

Chronic inflammation’s role in cancer is further exemplified by its ability to induce genomic instability through oxidative stress-induced DNA damage (77, 78). This damage can accumulate over time, fostering an environment ripe for the acquisition of oncogenic mutations that drive carcinogenesis. Therefore, the relationship between diet, inflammation, and cancer is multifaceted, extending from the systemic level of metabolism to cellular mechanisms and ultimately to the integrity of the genome. Understanding these connections can lead to the development of dietary interventions designed to reduce inflammation and oxidative stress, potentially preventing or delaying the onset of cancer (**Table 1**).

**Table 1:** Major endocrine-related mechanisms linking nutrition to breast cancer.

Mechanism	Key dietary drivers	Representative biomarkers	Key biological effects	Clinical implications	Refs.
Estrogen Signaling	Phytoestrogens; dietary fiber; cruciferous vegetables; high-fat diet	Circulating estradiol, estrone; SHBG; 2-OHE1/16α-OHE1 ratio	Modulates aromatase activity; alters ERα/ERβ activation; regulates cell proliferation and apoptosis	Prediction of endocrine therapy efficacy and resistance in ER-positive breast cancer	(43, 46, 49, 58)
Insulin/IGF Axis	High glycemic load; refined carbohydrates; excess saturated fat; hypercaloric diets	Fasting insulin; C-peptide; HOMA-IR; circulating IGF-1; IGFBP-3	Activates IR/IGF-1R; stimulates PI3K/AKT/mTOR and RAS/MAPK cascades; promotes anabolic metabolism	Identification of metabolically high-risk individuals; correlates with treatment resistance and poorer survival	(29, 31, 33, 42)
Adipokine Signaling	Excessive caloric intake; high omega-6/omega-3 ratio; Western dietary patterns	Serum leptin; adiponectin; leptin/adiponectin ratio; resistin	Induces hyperleptinemia and hypoadiponectinemia; drives angiogenesis, chronic inflammation, and metabolic reprogramming	Evaluation of obesity-associated breast cancer risk; prognostic monitoring in postmenopausal women	(19–21, 27)
Inflammatory & Oxidative Stress	Saturated/trans fats; refined sugars; low antioxidant intake; Mediterranean diet (protective)	CRP; IL-6; TNF-α; 8-OHdG; MDA; NF-κB activity markers	Activates NF-κB and STAT3; induces ROS-mediated DNA damage; promotes EMT and immunosuppressive TME	Assessment of systemic inflammatory burden; predicts poorer response to neoadjuvant therapy and immunotherapy	(60, 64, 68, 75)

Collectively, inflammation and oxidative stress represent fundamental biological processes linking nutritional exposures with endocrine dysregulation and breast cancer progression (72). Rather than functioning as isolated mechanisms, they interact closely with estrogen signaling, insulin resistance, and adipokine imbalance to establish a complex tumor-promoting microenvironment (68). The four axes—adipokine dysregulation, insulin/IGF signaling, estrogen metabolism, and inflammatory-oxidative stress—form an interconnected network in which nutritional factors operate synergistically to shape breast cancer risk and outcomes. This integrated perspective provides a mechanistic foundation for understanding the clinical relevance of inflammatory and oxidative biomarkers discussed in the subsequent sections and supports the development of biomarker-guided nutritional interventions aimed at improving breast cancer prevention and prognosis (55, 68).

## 3 Nutritional biomarkers of breast cancer risk and prognosis

Nutritional biomarkers can be classified through two complementary frameworks that serve distinct but interconnected purposes. The first framework—functional classification (5, 17, 46) (recovery, concentration, replacement, predictive biomarkers)—organizes biomarkers according to what they measure and their biological level of inference. The second framework—molecular classification (circulating vitamins, fatty acid biomarkers, metabolomic biomarkers, adipokines, inflammatory biomarkers)—organizes biomarkers according to their chemical nature and analytical platform. These two systems are not mutually exclusive; rather, they represent orthogonal dimensions of classification. For example, serum folate is both a concentration biomarker (functional classification) and a circulating vitamin biomarker (molecular classification) (37, 39, 50, 54). Similarly, the leptin-to-adiponectin ratio functions as both a replacement biomarker and an adipokine biomarker. Composite metabolomic signatures span multiple functional categories simultaneously, serving as concentration, replacement, and predictive biomarkers depending on the analytical approach and clinical question (45, 60, 77). Understanding this dual classification is essential for selecting appropriate biomarkers for specific clinical applications and for interpreting their biological significance.

A critical but frequently overlooked barrier to clinical translation is the influence of pre-analytical confounding factors on nutritional biomarker measurements (65, 66). These factors can substantially affect biomarker concentrations independently of the biological phenomenon of interest, introducing measurement error and reducing the reproducibility of findings across studies. Key pre-analytical variables include: (i) sampling time and circadian variation—many nutritional biomarkers exhibit diurnal rhythms (e.g., cortisol, insulin, leptin), and blood collection at different times of day can yield markedly different values (74); (ii) fasting status—non-fasting samples can dramatically alter levels of triglycerides, glucose, insulin, and certain vitamins, making comparisons between fasting and non-fasting cohorts unreliable; (iii) specimen storage conditions—prolonged storage at suboptimal temperatures, repeated freeze-thaw cycles, and variations in anticoagulant type can degrade labile metabolites and compromise biomarker integrity (75, 77); (iv) racial and ethnic disparities—genetic polymorphisms in metabolic enzymes, differences in dietary patterns, body composition, and gut microbiome composition all contribute to substantial inter-population variability in baseline biomarker levels, meaning reference ranges established in Western populations may not be generalizable; and (v) seasonal variation—biomarkers such as vitamin D exhibit pronounced seasonal fluctuations related to sunlight exposure, which can confound cross-sectional comparisons. Standardization of pre-analytical protocols is therefore an essential prerequisite for reliable biomarker measurement and clinical implementation (58, 70).

### 3.1 Circulating nutrient biomarkers

Nutritional status, as reflected by the circulating levels of specific nutrients, offers an objective metric for assessing an individual’s health and is increasingly significant in epidemiological studies exploring the nexus between diet and the risk of developing breast cancer. Compared with traditional dietary questionnaires, circulating nutrient biomarkers provide a more direct assessment of nutrient exposure by integrating dietary intake with absorption efficiency, metabolism, and physiological utilization. Consequently, these biomarkers are less susceptible to recall bias and inter-individual variation in dietary reporting, making them valuable tools for investigating diet–disease relationships and improving the accuracy of nutritional risk assessment. As advances in analytical technologies continue to improve the sensitivity and reproducibility of biomarker detection, circulating nutrients are increasingly recognized as promising indicators for breast cancer prevention, prognosis evaluation, and personalized nutritional interventions.

Folate, a water-soluble B vitamin integral to one-carbon metabolism, is pivotal in key cellular processes such as DNA synthesis (79), repair, and methylation. Mounting evidence suggests that folate’s influence on breast cancer risk is multifaceted and may vary by menopausal status, alcohol consumption, and genetic polymorphisms affecting folate metabolic enzymes (80). While some prospective studies indicate an inverse association between circulating folate levels and breast cancer risk—particularly among women with elevated alcohol intake (81)—the inconsistencies in findings underscore the intricate nature of this relationship. The biphasic effect of folate on cancer—wherein optimal levels might mitigate initial carcinogenesis (82), yet excessive intake could potentiate the growth of established tumors—emphasizes the necessity for a nuanced approach, considering both the timing and dosage of folate supplementation in any preventative strategies (83). Beyond maintaining genomic stability, folate availability influences epigenetic regulation through DNA methylation, thereby affecting the expression of genes involved in cell-cycle control and tumor suppression. This dual biological role may partly explain the conflicting epidemiological findings and suggests that circulating folate concentrations should be interpreted within the broader context of metabolic status and disease stage rather than as an isolated nutritional indicator.

Vitamin D, celebrated for its broad-spectrum effects including the regulation of cell proliferation, differentiation, apoptosis, and angiogenesis, is another nutrient subjected to intense scrutiny concerning its role in breast cancer prognosis (84). Ecological studies revealing a lower incidence of breast cancer in regions with abundant sunlight (and thus higher vitamin D biosynthesis) provide preliminary support for its preventative potential (85). However, the connection between serum 25-hydroxyvitamin D levels and breast cancer risk remains inconsistent across studies. Nonetheless, certain meta-analyses have noted a modest inverse association, especially in postmenopausal women (86, 87). The impact of vitamin D on survival outcomes is more firmly established, with higher levels at diagnosis correlating with improved survival in breast cancer patients (88). Increasing evidence also indicates that vitamin D participates in immune regulation and maintenance of tissue homeostasis by influencing inflammatory signaling and cellular differentiation. These pleiotropic effects suggest that serum vitamin D concentrations may reflect not only nutritional status but also broader endocrine and immunometabolic conditions that influence breast cancer progression. Nevertheless, variations in sunlight exposure, geographical latitude, supplementation practices, and assay methodologies continue to complicate comparisons across epidemiological studies.

Carotenoids, a class of lipid-soluble pigments found in fruits and vegetables, have garnered attention for their antioxidant properties and their hypothesized role in cancer prevention (89). These compounds, including α-carotene, β-carotene, and lycopene, engage in neutralizing singlet oxygen, scavenging free radicals, and modulating immune function. Pooled analyses demonstrate an inverse association between serum carotenoid levels and the risk of breast cancer, with especially robust results for α-carotene and β-carotene (90). The protective effects of carotenoids are notably pronounced for tumors that are estrogen receptor-negative, hinting at distinct etiologies for different subtypes of breast cancer. In addition to their antioxidant capacity, carotenoids may contribute to the preservation of cellular redox balance and regulation of inflammatory responses, thereby limiting oxidative DNA damage and maintaining genomic integrity. These biological properties provide mechanistic support for the observed epidemiological associations while emphasizing that circulating carotenoid levels may serve as indicators of both dietary quality and systemic antioxidant capacity.

Contemporary research has expanded on this foundation, elucidating the molecular interactions between circulating nutrients and breast cancer biology. Vitamin D, for instance, has been shown to influence the expression of microRNAs that are involved in epithelial-mesenchymal transition (91), a key process in cancer metastasis. Folate status may similarly impinge upon breast cancer risk through epigenetic alterations, especially the DNA methylation patterns that govern gene expression (92). The relationship between carotenoids and breast cancer risk is further suggested to be modified by factors (89, 93) such as smoking status, body mass index, and menopausal status, which underscore the complexity of nutritional epidemiology. These mechanistic insights not only reinforce the plausibility of observed associations between circulating nutrient biomarkers and breast cancer but may also elucidate certain discrepancies in epidemiological research findings. Collectively, current evidence indicates that no single circulating nutrient is likely to provide sufficient predictive value on its own. Instead, integrating multiple nutritional biomarkers with endocrine, metabolic, and inflammatory indicators may provide a more comprehensive characterization of individual risk profiles (78, 89). Such multidimensional approaches are increasingly aligned with precision nutrition and biomarker-guided breast cancer management, offering opportunities for improved risk stratification, prognosis prediction, and individualized preventive strategies while highlighting the need for standardized biomarker measurement and prospective validation in diverse populations.

### 3.2 Fatty acid biomarkers

Fatty acid biomarkers occupy a unique position in nutritional biomarker research because they integrate dietary intake with endogenous metabolism and reflect both short-term and long-term nutritional exposure (58, 78). Circulating fatty acid profiles—particularly the omega-3/omega-6 polyunsaturated fatty acid (PUFA) ratio (53, 54, 67)—serve as concentration biomarkers reflecting dietary lipid intake patterns, while also functioning as replacement biomarkers indicative of inflammatory balance and membrane fluidity (70).

The omega-3/omega-6 PUFA ratio has been extensively investigated in relation to breast cancer risk. Higher circulating levels of omega-3 fatty acids (EPA, DHA, and alpha-linolenic acid) are generally associated with reduced breast cancer risk, particularly in postmenopausal women, whereas elevated omega-6 fatty acids (particularly arachidonic acid) (69, 84) may promote tumorigenesis through pro-inflammatory eicosanoid production. Conjugated linoleic acid (CLA), a group of isomers found primarily in dairy and ruminant meat products, has demonstrated anti-proliferative and pro-apoptotic effects in preclinical models, although epidemiological evidence regarding its association with breast cancer risk remains inconsistent (85, 86).

Beyond individual fatty acids, comprehensive lipidomic profiles have revealed that specific lipid species—including certain phospholipids, sphingolipids, and ceramides—are associated with breast cancer risk and prognosis (87). These lipid biomarkers reflect alterations in membrane composition, intracellular signaling, and mitochondrial function that occur early in carcinogenesis. Importantly, because fatty acid biomarkers are directly modulated by dietary intake, they represent particularly attractive targets for nutritional intervention studies (58, 66, 80).

### 3.3 Metabolomic signatures

Metabolomics, which entails the detailed examination of small-molecule metabolites, has rapidly emerged as a robust analytical tool in oncology, particularly for understanding the multifaceted biology of breast cancer (15, 42). Unlike individual nutrient biomarkers, metabolomic profiling captures the integrated downstream consequences of dietary intake, endogenous metabolism, genetic susceptibility, endocrine regulation, and environmental exposures (35, 86). This systems-level approach provides a comprehensive snapshot of an individual’s metabolic phenotype, reflecting both physiological adaptation and pathological alterations. Consequently, metabolomics has become an increasingly valuable platform for identifying biomarkers associated with breast cancer risk, disease progression, and therapeutic response.

Several prospective studies (94, 95) have begun to unveil the metabolomic profiles that precede the development of breast cancer. These investigations have delineated a landscape in which alterations in enzymes and intermediary metabolites reshape the baseline metabolic state, manifesting as coordinated changes in lipid metabolism, amino acid turnover, energy production, and redox homeostasis (88, 96). Rather than representing isolated metabolic abnormalities, these alterations reflect extensive metabolic reprogramming that accompanies early carcinogenesis. Such metabolomic signatures therefore possess considerable potential not only as predictive biomarkers for disease risk but also as indicators of the biological pathways linking nutrition with breast cancer development (27, 47).

One noteworthy instance in the arena of metabolomics is the ongoing research helmed by Kristal and colleagues (96), which explores metabolome-based approaches for breast cancer risk prediction. This pioneering study seeks to determine whether metabolic signatures associated with habitual caloric intake can prospectively predict breast cancer development while simultaneously identifying circulating metabolites with potential utility as objective nutritional biomarkers. Encompassing longitudinal follow-up of approximately 30,000 participants (4, 17, 55), this large-scale investigation represents one of the most comprehensive efforts to integrate nutritional epidemiology with metabolomics (46, 80). Its findings may substantially improve the precision of dietary exposure assessment while facilitating the development of individualized risk prediction models based on metabolic phenotypes rather than self-reported dietary intake alone (81, 88).

One of the most significant recent advances in metabolomic breast cancer research is the identification of subtype-specific metabolic signatures associated with differential prognosis. These signatures vary substantially across the major molecular subtypes of breast cancer, reflecting distinct metabolic dependencies and tumor biology:

Triple-negative breast cancer (TNBC) exhibits the most aggressive metabolic phenotype, characterized by elevated glycolysis (46), enhanced glutaminolysis (88), and profound mitochondrial metabolic reprogramming (90). Poor prognosis in TNBC is consistently associated with signatures of increased acylcarnitine species (reflecting impaired fatty acid β-oxidation and mitochondrial dysfunction), elevated branched-chain amino acid (BCAA) catabolites (92, 95), and increased kynurenine pathway activation (indicating immune suppression through tryptophan depletion). Conversely, favorable prognosis in TNBC has been linked to higher levels of certain glycerophospholipids and polyunsaturated fatty acid metabolites that may reflect preserved membrane integrity and reduced inflammatory signaling (94, 95). Notably, TNBC metabolomic signatures show the strongest prognostic value among all subtypes, likely because this subtype lacks targeted therapies and metabolic vulnerabilities represent potential therapeutic targets (96).

In luminal-like (ER-positive) breast cancer, prognostic metabolomic signatures are more closely tied to endocrine and lipid metabolism (97). Poor prognosis is associated with elevated steroid hormone metabolites, increased choline and phosphocholine levels (reflecting enhanced membrane biosynthesis and proliferation), and altered bile acid profiles. Good prognosis in luminal tumors has been linked to higher circulating levels of phytoestrogen metabolites (enterolactone, equol) and favorable fatty acid profiles (98), consistent with the hormone-dependent nature of this subtype. The prognostic value of metabolomic signatures in luminal breast cancer is particularly relevant for predicting endocrine therapy response and identifying patients at risk for late recurrence (99).

HER2-enriched breast cancer displays metabolic signatures reflecting enhanced anabolic metabolism and receptor tyrosine kinase-driven metabolic reprogramming (100). Poor prognosis is associated with elevated nucleotide synthesis intermediates, increased glucose metabolism byproducts, and altered phospholipid profiles indicative of rapid cellular proliferation (65, 88, 91). Metabolomic signatures in HER2-positive disease may also have predictive value for anti-HER2 therapy response (87), as metabolic adaptation is closely linked to therapeutic resistance mechanisms.

Across all subtypes, certain metabolomic features emerge as consistent predictors of poor prognosis: elevated lactate (reflecting the Warburg effect), increased polyamine metabolites (indicating enhanced proliferation), and altered acylcarnitine profiles (suggesting mitochondrial dysfunction) (64, 82, 97). Conversely, higher levels of omega-3 fatty acid metabolites, certain carotenoid breakdown products, and favorable tryptophan/kynurenine ratios are generally associated with better outcomes (92, 95). These subtype-specific metabolomic signatures not only provide prognostic information but also reveal metabolic vulnerabilities that could be exploited through targeted nutritional interventions.

Lipidomics, a specialized branch of metabolomics, has further revealed characteristic lipid alterations associated with breast cancer. Studies have identified changes in phospholipids, sphingolipids, and carnitine esters (97, 98) during the pre-diagnostic phase among women who subsequently develop breast cancer. These metabolic alterations suggest disturbances in membrane remodeling, intracellular signaling, mitochondrial energy metabolism (55), and lipid utilization that may occur long before clinical diagnosis. Importantly, because lipid metabolism is closely regulated by nutritional status, obesity, and endocrine signaling, these lipidomic biomarkers provide an important mechanistic link between metabolic health and breast carcinogenesis. Integrating lipidomic profiles with established clinical risk factors may therefore improve early risk stratification and enhance the identification of individuals who could benefit from preventive interventions (78).

The continuous advancement of high-resolution metabolomic technologies has enabled increasingly comprehensive characterization of metabolic networks involved in breast cancer. Alterations in mitochondrial metabolism (99), reflected by changes in acylcarnitine profiles, have emerged as important indicators of disrupted cellular bioenergetics and breast cancer risk. These observations suggest impaired fatty acid β-oxidation (100), altered mitochondrial function, and reduced metabolic flexibility during tumor development. In parallel, abnormalities in tryptophan metabolism, particularly activation of the kynurenine pathway, have been increasingly associated with breast cancer initiation and progression through interactions with immune regulation and inflammatory signaling. Collectively, these findings illustrate that metabolomic biomarkers extend beyond simple indicators of nutrient exposure and instead reflect dynamic interactions among metabolism, endocrine function, and the tumor microenvironment.

The integration of metabolomic data with other omics technologies—including genomics and proteomics (101)—has further expanded our understanding of breast cancer biology. Multi-omics analyses enable simultaneous evaluation of metabolic alterations alongside genetic, transcriptomic, and proteomic changes, thereby providing a more comprehensive view of disease heterogeneity and metabolic adaptation (102). Such integrative strategies may facilitate the identification of previously unrecognized metabolic vulnerabilities while improving biomarker discovery and personalized risk prediction (103). As metabolomic technologies continue to mature, standardization of analytical platforms, validation across diverse populations, and integration with traditional clinical indicators will be essential for translating metabolomic signatures into routine clinical practice (104). Ultimately, combining metabolomic biomarkers with circulating nutrient, endocrine, and inflammatory biomarkers may provide a more accurate framework for precision nutrition and individualized breast cancer prevention.

### 3.4 Inflammatory and adipokine biomarkers

In the complex tapestry of factors linking nutrition to breast cancer pathogenesis, circulating biomarkers of inflammation and adipose tissue activity have emerged as pivotal functional indicators. C-reactive protein (CRP), an acute-phase reactant synthesized by the liver under the influence of IL-6, is a cardinal measure of systemic inflammation and has implicative ties with the risk of breast carcinogenesis (102). A wealth of meta-analyses (103–105) have delineated an association between elevated CRP levels and an escalated risk of breast cancer, particularly amongst postmenopausal women and those bearing tumors that are hormone receptor-positive. However, the relationship between CRP and breast cancer prognosis is less uniform, with some studies indicating that pre-treatment CRP elevations are indicative of poorer survival outcomes.

Adipokines, cytokines secreted by adipose tissue, serve as intermediaries in the intricate nexus between obesity and breast cancer risk (106). Leptin, an adipokine endowed with pro-inflammatory and pro-angiogenic attributes, shows a robust positive correlation with body fat mass, with some epidemiological studies identifying high leptin levels as a harbinger of increased breast cancer risk (107). This risk stratification appears dependent on factors such as menopausal status and tumor characteristics, with postmenopausal women and hormone receptor-positive tumor cases exhibiting more pronounced associations. Adiponectin, in contrast, is an insulin-sensitizing adipokine characterized by anti-inflammatory and anti-proliferative properties, which has consistently demonstrated an inverse association with breast cancer risk across most studies (108). The ratio of leptin to adiponectin has been proposed as a potentially more insightful biomarker (109), encapsulating the delicate equilibrium between pro-carcinogenic and anti-carcinogenic adipose tissue signals.

Recent investigative advancements (110–112) have expanded our grasp of the role of inflammatory and adipokine biomarkers, surpassing their utility in mere risk prediction to their potential in modulating treatment responses. For instance, elevated pre-treatment levels of CRP and IL-6 have been correlated with the diminished efficacy of neoadjuvant chemotherapy in breast cancer patients, possibly signifying an immunosuppressive tumor microenvironment that hinders treatment efficacy. Likewise, elevated leptin levels have been implicated in resistance to endocrine therapies, suggesting that adipokine signaling could emerge as a therapeutic target to bolster treatment efficacy. A recent multicenter real-world study (113) underscored the predictive value of inflammatory and nutritional markers in achieving pathological complete response to neoadjuvant therapy in HER2-positive breast cancer, highlighting their clinical application in treatment selection and monitoring. These findings stress the imperative of considering nutritional and inflammatory status not just for risk assessment but also for optimizing cancer therapies (**Table 2**).

**Table 2:** Selected nutritional biomarkers and their clinical significance in breast cancer.

Biomarker category	Representative biomarkers	Association with breast cancer risk	Prognostic value (subtype-specific where available)	Refs.
Circulating Vitamins	Folate; 25-hydroxyvitamin D [25(OH)D]; α-/β-carotene; lycopene	Inverse association for vitamin D and carotenoids; folate risk is dose- and timing-dependent	Higher 25(OH)D at diagnosis consistently correlates with improved overall and disease-free survival	(81, 87, 88, 90)
Fatty Acid Biomarkers	Omega-3/omega-6 PUFA ratio; conjugated linoleic acid (CLA)	Higher omega-3 levels linked to reduced risk, particularly in postmenopausal women	Evidence remains limited and inconsistent; lipidomic subclasses show emerging subtype-specific trends	(89, 97)
Metabolomic Signatures	Acylcarnitines; kynurenine; branched-chain amino acid (BCAA) catabolites; glycerophospholipids	Emerging evidence supports predictive value years before clinical diagnosis	Poor prognosis: elevated acylcarnitines and kynurenine (TNBC); Favorable prognosis: higher glycerophospholipids and omega-3 metabolites (Luminal/HER2-enriched)	(94, 95, 97, 98)
Adipokines	Leptin; adiponectin; leptin/adiponectin ratio	Leptin positively associated (especially in postmenopausal ER+ disease); adiponectin inversely associated	Elevated leptin and low adiponectin correlate with aggressive tumor features, endocrine resistance, and worse recurrence-free survival	(107–109)
Inflammatory Markers	CRP; IL-6; TNF-α	Elevated CRP consistently associated with increased risk, particularly in postmenopausal women	Pre-treatment CRP/IL-6 elevation predicts poorer response to neoadjuvant chemotherapy and reduced survival	(102–104)

### 3.5 Clinical translation and future perspectives of nutritional biomarkers

Although substantial progress has been achieved in identifying nutritional biomarkers associated with breast cancer, translating these discoveries into routine clinical practice remains challenging. Most currently available biomarkers demonstrate moderate predictive performance when evaluated individually, reflecting the multifactorial nature of breast cancer development. Consequently, increasing attention has shifted toward integrated biomarker models that combine circulating nutrients, metabolic signatures, inflammatory mediators, adipokines, and endocrine indicators to improve predictive accuracy.

The rapid development of high-throughput metabolomics, lipidomics, proteomics, and machine learning has created new opportunities for multidimensional biomarker discovery. Rather than relying on single biomarkers, future predictive models are expected to incorporate multiple biological layers alongside conventional clinicopathological variables, thereby providing more comprehensive characterization of individual metabolic risk profiles. Such approaches may facilitate earlier identification of high-risk populations, improve prognostic stratification, and support personalized nutritional interventions during cancer prevention and treatment.

Despite these advances, several critical barriers to clinical translation remain. First, the absence of standardized pre-analytical and analytical protocols—encompassing sampling conditions, specimen handling, assay platforms, and reference ranges—undermines the comparability of findings across studies. Second, most biomarker discoveries have been made in retrospective, single-center cohorts with predominantly Western populations, limiting generalizability. Third, the dynamic nature of nutritional biomarkers—their sensitivity to recent diet, circadian rhythms, and acute physiological changes—complicates their use as stable prognostic indicators (54, 103). Fourth, single biomarkers generally lack sufficient specificity and sensitivity for clinical decision-making, necessitating the development of composite panels. Addressing these barriers through international standardization efforts, large prospective multicenter validation studies, and the development of robust multi-biomarker algorithms will be essential for realizing the clinical potential of nutritional biomarkers in breast cancer care.

## 4 Integration and application of biomarkers in prognosis prediction

### 4.1 Development of biomarker-based prediction models

The integration of multiple biomarkers into comprehensive prediction models holds significant potential for more accurate risk stratification and prognosis assessment in breast cancer. Traditional prognostic factors, such as tumor stage, grade, and receptor status, while valuable, do not fully capture the biological heterogeneity of breast cancer or its complex interactions with host factors. The incorporation of nutritional and endocrine biomarkers can significantly enhance prognostic models by reflecting the systemic metabolic and hormonal influences on tumor behavior. Rather than functioning independently, these biomarkers provide complementary information regarding metabolic status, inflammatory activity, endocrine regulation, and nutritional exposure, thereby enabling a more comprehensive characterization of the biological processes underlying tumor progression. This integrated approach facilitates a more nuanced understanding of disease heterogeneity and supports the development of personalized treatment strategies that are more effective and less harmful to patients.

The concept of biomarker integration also reflects a broader transition from conventional clinicopathological prediction toward systems biology-based precision oncology. As increasing evidence demonstrates that breast cancer progression is influenced by dynamic interactions between tumor cells and host metabolism, combining biomarkers from multiple biological pathways has become essential for improving predictive accuracy. Nutritional biomarkers, metabolomic signatures, inflammatory mediators, and endocrine indicators collectively provide complementary insights into both tumor biology and host physiological status, thereby extending prognostic assessment beyond traditional tumor-centered parameters.

The development of biomarker-based prediction models in the realm of breast cancer research involves several critical steps. Initially, promising biomarkers are identified through preclinical studies, epidemiological investigations, and high-throughput omics technologies, such as genomics, transcriptomics, and proteomics. These candidates are then subjected to rigorous validation in patient cohorts characterized for specific clinical endpoints, which may include survival rates, recurrence, or response to therapy. Subsequent to validation, multivariate statistical models are constructed to integrate these biomarkers with traditional factors (114). Techniques such as Cox proportional hazards regression are often employed for time-to-event data, allowing for the assessment of the relationship between different biomarkers and patient outcomes. Increasingly, variable selection approaches and machine-learning algorithms are being incorporated to manage the high dimensionality of biomarker datasets and to identify combinations of biomarkers that provide superior predictive performance. These models are essential in formulating individualized prognostic estimates that can ultimately guide clinical decision-making.

Rigorous internal and external validation is necessary to ensure the performance and broad applicability of these models before they can be implemented in clinical practice. This process, as depicted in **Figure 4**, is crucial in establishing the reliability and robustness of the prediction models. Without such validation, the models risk providing misleading prognostic information, which could adversely affect patient care. Equally important are standardized biomarker measurement protocols, reproducible analytical platforms, and validation across geographically and ethnically diverse populations to ensure that prediction models remain reliable in different clinical settings.

**Figure 4:**
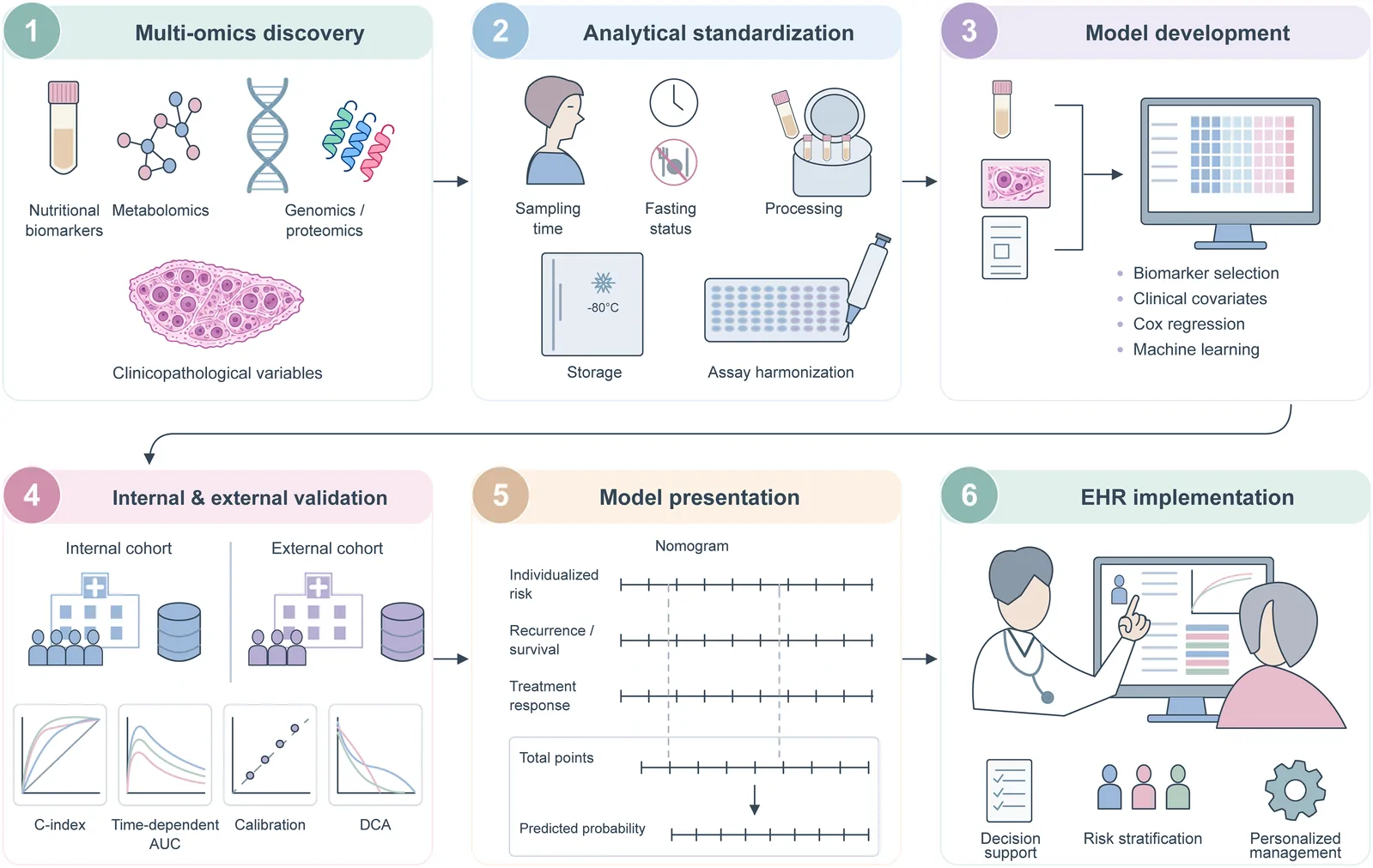
Iterative translational pipeline for biomarker-guided prognostic models, encompassing multi-omics discovery, analytical standardization, internal/external validation (C-index, AUC, calibration, DCA), nomogram construction, and EHR clinical implementation.

In recent years, nomograms have emerged as a preferred tool for prognostic predictions, providing a visual representation of mathematical models. These nomograms enable individual patient risk assessments (115), where points are assigned for various prognostic variables and summed to generate individualized estimates of clinical outcomes. Compared with conventional staging systems, nomograms permit simultaneous incorporation of clinicopathological characteristics and molecular biomarkers, thereby improving individualized risk prediction. Although the available literature provides limited details, recent studies such as those by Lin and others (114) on nutritional and metabolic biomarkers in breast cancer risk highlight a growing interest in enhancing predictive models with metabolic components, which could offer a more holistic view of the patient’s cancer risk profile. Future nomograms incorporating circulating nutrient biomarkers, adipokines, inflammatory markers, and metabolomic signatures may further improve predictive discrimination while supporting personalized nutritional management.

The advent of artificial intelligence (AI) in predictive modeling has marked a significant turning point in breast cancer prognosis. For example, the PreciseDx Breast test (PDxBR™) (116–118) exemplifies a novel approach that leverages AI-powered analysis of histopathological images in conjunction with clinical variables to predict the likelihood of early breast cancer recurrence. This assay employs machine-learning algorithms to extract quantifiable morphological features from standard hematoxylin and eosin (H&E)-stained tissue sections, yielding a risk score predictive of recurrence within a six-year timeframe. In a similar vein, the development of “BlurryScope” (119), a compact AI-powered microscope integrating simple optical hardware with deep-learning algorithms, demonstrates the potential of computational techniques to augment the accessibility and precision of biomarker assessment. Beyond image analysis, AI is increasingly being applied to integrate heterogeneous datasets encompassing imaging, clinical information, nutritional biomarkers, metabolomics, and molecular profiling. Such multimodal analytical frameworks may substantially improve prediction accuracy while facilitating real-time clinical decision support, particularly in the context of precision oncology.

Despite these promising advances, several challenges remain before biomarker-based prediction models can be routinely implemented in clinical practice. Biological heterogeneity, differences in laboratory methodologies, limited external validation, and the lack of universally accepted biomarker thresholds continue to restrict clinical translation. Furthermore, predictive models should be evaluated not only according to statistical performance but also with respect to interpretability, cost-effectiveness, and clinical utility. Addressing these issues will be essential for integrating biomarker-guided prediction into routine breast cancer management.

In conclusion, the development of biomarker-based prediction models represents a critical avenue for advancing breast cancer prognosis and personalized care. By integrating nutritional biomarkers with endocrine, inflammatory, metabolomic, and clinicopathological information, these models offer a more comprehensive representation of disease biology than conventional prognostic systems alone. Coupled with advances in artificial intelligence and multi-omics technologies, biomarker-driven prediction models are expected to improve individualized risk assessment, optimize therapeutic decision-making, and support precision nutrition strategies throughout the continuum of breast cancer prevention, treatment, and survivorship.

### 4.2 Validation and performance metrics

In the realm of medical research, the evaluation of predictive models requires rigorous assessment of multiple performance metrics to determine their reliability, clinical applicability, and potential for implementation. A robust prognostic model should not only accurately distinguish between patients with different clinical outcomes but also provide predictions that are reproducible across diverse patient populations. As biomarker-based models increasingly integrate nutritional, endocrine, inflammatory, and metabolomic indicators, comprehensive validation has become an indispensable prerequisite for clinical translation.

Each performance metric evaluates a distinct and complementary dimension of predictive utility, and understanding these differences is essential for interpreting the clinical value of a prediction model:

The C-index (concordance index) measures the overall discriminative ability of a model—specifically, the probability that, for any randomly selected pair of patients, the patient who experiences the event first will have a higher predicted risk. In essence, it evaluates how well the model ranks patients by risk level. A C-index of 0.5 represents no better than chance, while 1.0 represents perfect discrimination. In breast cancer prognostic models (87, 95), C-index values generally range from 0.70 to 0.85. The unique clinical value of the C-index lies in its ability to quantify overall model discrimination across the entire follow-up period with a single number, making it convenient for comparing different models (110). However, it does not reveal whether discrimination varies at different time points or across risk strata.

Time-dependent AUC (area under the receiver operating characteristic curve) extends the concept of discrimination to specific time points, evaluating how well a model distinguishes between patients who will and will not experience an event by a given time (e.g., 3-year or 5-year survival) (120). Unlike the C-index, which provides an overall measure, time-dependent AUC reveals the temporal dynamics of predictive performance—whether the model is more accurate at earlier versus later follow-up times. This is particularly valuable in breast cancer, where prognostic factors may have differential importance at different disease stages (103, 105, 110). For example, tumor size may strongly predict early recurrence but have diminishing value for late recurrence, whereas metabolic biomarkers may gain prognostic importance over time. Time-dependent AUC thus provides a more nuanced understanding of when and how a model adds clinical value.

Calibration assesses the agreement between predicted probabilities and observed outcomes—whether patients with a predicted 30% risk of recurrence actually have approximately a 30% observed recurrence rate. This is fundamentally different from discrimination: a model can discriminate well (C-index = 0.80) yet be poorly calibrated (systematically overestimating or underestimating risk) (64, 85, 92). Calibration is typically evaluated graphically using calibration plots, where predicted probabilities are plotted against observed event rates, with perfect calibration represented by the diagonal reference line (y = x) (88). The clinical importance of calibration cannot be overstated: if a model is well-discriminated but poorly calibrated, it may systematically misclassify patients into incorrect risk categories, leading to inappropriate treatment decisions. For nutritional biomarker models, calibration is particularly critical because biomarker distributions can vary substantially across populations, and a model calibrated in one cohort may be miscalibrated when applied to another.

Decision Curve Analysis (DCA) (27, 75) evaluates the clinical utility of a model by quantifying the net benefit of using the model for decision-making across a range of risk thresholds. Net benefit is calculated as the number of true positives minus the weighted number of false positives, expressed per 100 patients. Unlike discrimination and calibration, which are purely statistical measures, DCA directly addresses the clinical question: does using this model lead to better patient outcomes than either treating everyone or treating no one? DCA curves plot net benefit against threshold probability, allowing clinicians to determine at what risk threshold the model provides the greatest clinical value (28, 29). For nutritional biomarker-guided interventions, DCA is especially informative because it helps identify the optimal risk threshold at which the benefits of intervention (e.g., dietary modification, intensified monitoring) outweigh the harms (e.g., unnecessary intervention, patient anxiety) (54, 56). A model with good discrimination and calibration may still have limited clinical utility if the net benefit is marginal across clinically relevant threshold ranges.

Together, these four metrics—C-index, time-dependent AUC, calibration, and DCA—provide a comprehensive evaluation of prognostic model performance. C-index and time-dependent AUC answer “how well does the model rank patients?”; calibration answers “how accurate are the predicted probabilities?”; and DCA answers “does using the model actually improve clinical decisions?” A model that performs well across all four dimensions is most likely to be clinically useful. For nutritional biomarker models, achieving strong performance across all dimensions is particularly challenging because of the dynamic nature of metabolic biomarkers and the influence of pre-analytical factors, underscoring the importance of rigorous validation and standardization (29, 72, 95).

Within the scope of internal validation, resampling techniques such as bootstrapping and cross-validation are instrumental in estimating model performance within the original dataset, thereby minimizing overfitting and improving reproducibility. Conversely, external validation, in which prediction models are evaluated using independent patient cohorts, remains the gold standard for determining generalizability and real-world applicability (101, 107). Because biomarker distributions may differ substantially across populations with varying ethnic backgrounds, dietary habits, healthcare systems, and disease characteristics, successful external validation is particularly important for nutrition-related prediction models. Illustratively, recent studies (121, 122) investigating the CD155/CD226/TIGIT/CD96 immune checkpoint axis have developed validated multi-biomarker prognostic models with C-indices ranging from 0.772 to 0.822 for survival prediction. These models further demonstrated favorable performance through decision curve analysis, supporting their potential clinical utility (122).

When nutritional and endocrine biomarkers are incorporated into prediction models, additional methodological challenges arise that require specific consideration. Unlike genomic biomarkers, which generally remain relatively stable throughout life, nutritional biomarkers are inherently dynamic and may fluctuate according to recent dietary intake, seasonal variation, physical activity, body weight, medication use, and other lifestyle factors (123). Consequently, the timing of biospecimen collection, repeated biomarker measurements, and longitudinal assessment become particularly important during model development. Furthermore, because nutritional exposures differ substantially among geographic regions and populations, prediction models developed within one dietary context may exhibit reduced performance when applied elsewhere. These characteristics emphasize the necessity of standardized sample collection procedures, harmonized laboratory methodologies, and multicenter validation before widespread clinical implementation.

Recent methodological advances have begun to address these challenges. Standardized protocols for biomarker quantification, improved quality-control procedures, and longitudinal cohort designs incorporating repeated biomarker measurements have enhanced the reliability and reproducibility of nutrition-based prediction models (123). At the same time, advances in high-throughput analytical technologies and machine-learning algorithms have enabled more robust integration of heterogeneous biomarker data while reducing the influence of measurement variability. These developments are expected to improve both predictive accuracy and the practical applicability of biomarker-based prognostic models.

Overall, rigorous validation remains fundamental for translating biomarker-based prediction models from research settings into clinical practice. Reliable discrimination, accurate calibration, robust external validation, and demonstrated clinical utility collectively determine whether a prediction model can meaningfully improve patient management. As nutritional biomarkers become increasingly integrated with endocrine, inflammatory, metabolomic, and molecular data, standardized validation frameworks will play a central role in ensuring that these models provide reproducible, clinically actionable information capable of supporting precision oncology and personalized nutritional intervention in breast cancer.

### 4.3 Clinical implementation and challenges

The translation of biomarker-based prediction models from research settings into routine clinical practice is accompanied by numerous scientific, technical, and operational challenges. Central to this process are pragmatic considerations including the availability and standardization of biomarker assays, the reproducibility of laboratory measurements, the complexity of interpreting prediction model outputs, and their seamless integration into existing clinical workflows (124). Because nutritional, endocrine, inflammatory, and metabolomic biomarkers originate from diverse analytical platforms, harmonization of sample collection, laboratory procedures, and quality-control standards is essential to ensure consistent and reproducible results across institutions. Without standardized methodologies, variability in biomarker measurement may substantially reduce model reliability and hinder widespread clinical adoption.

To facilitate implementation, the development of user-friendly digital platforms and the integration of prediction models into electronic health record systems are increasingly recognized as key priorities. Automated calculation of individualized risk scores, combined with intuitive visualization of prediction results, may substantially reduce clinicians’ workload while improving the accessibility and interpretability of biomarker information (125). Such digital decision-support systems may also facilitate continuous model updating as new biomarker evidence emerges, allowing prediction algorithms to evolve alongside advances in nutritional science, molecular oncology, and artificial intelligence. Equally important is the incorporation of validated biomarker-based prediction models into evidence-based clinical guidelines, accompanied by clear recommendations regarding biomarker testing, interpretation, and subsequent therapeutic decision-making.

The economic feasibility of biomarker-guided screening and intervention strategies represents another critical determinant of successful implementation. Although comprehensive biomarker panels inevitably increase initial diagnostic expenditures, these costs may be offset by improvements in treatment selection, avoidance of unnecessary therapies, and optimization of healthcare resource utilization (126). Patients identified as having low-risk biological profiles may avoid overtreatment and its associated toxicities, whereas individuals with high-risk metabolic or inflammatory phenotypes may benefit from intensified monitoring or targeted therapeutic interventions. Consequently, comprehensive cost-effectiveness analyses comparing biomarker-guided management with conventional clinical practice are essential to support healthcare policy development, reimbursement decisions, and equitable implementation across different healthcare systems.

Among the emerging applications of biomarker-guided medicine, nutritional and endocrine biomarkers possess particular promise for advancing personalized breast cancer management (127). Identification of distinct metabolic and endocrine phenotypes may assist clinicians in selecting patients who are most likely to benefit from individualized nutritional interventions, lifestyle modification programs, or therapies targeting metabolic pathways. Rather than considering nutrition solely as a preventive measure, the concept of metabolic phenotyping expands its role throughout the entire continuum of breast cancer care, including risk assessment, treatment optimization, survivorship management, and long-term prognosis. Such strategies reflect the growing convergence of precision oncology and precision nutrition, in which host metabolic characteristics are incorporated alongside tumor molecular profiles to guide individualized clinical decision-making.

Despite these promising developments, several barriers remain before biomarker-guided approaches can become routine components of clinical care. The biological variability of nutritional biomarkers, differences in dietary patterns across populations, and the dynamic influence of age, obesity, physical activity, medications, and comorbidities complicate interpretation of biomarker measurements. Furthermore, many proposed biomarkers have been validated primarily in retrospective or single-center studies, emphasizing the need for large prospective multicenter investigations involving diverse populations before widespread implementation. Continued efforts toward analytical standardization, external validation, and international consensus regarding biomarker thresholds will therefore be essential for ensuring clinical reliability.

The implementation of biomarker-guided strategies also raises important ethical, educational, and societal considerations. Equitable access to advanced biomarker testing should be ensured to avoid widening existing healthcare disparities. In addition, communication of biomarker-derived risk information requires careful interpretation, as inappropriate presentation of individualized risk estimates may lead to unnecessary anxiety or false reassurance. Successful implementation therefore depends not only on technological advances but also on multidisciplinary collaboration among oncologists, nutrition specialists, pathologists, laboratory scientists, bioinformaticians, and primary care providers. Continuous professional education and standardized reporting systems will further facilitate accurate interpretation and appropriate clinical application of biomarker information.

Overall, successful clinical translation of biomarker-based prediction models requires a comprehensive framework that integrates scientific rigor with practical implementation. Standardized laboratory procedures, robust external validation, digital clinical decision-support systems, evidence-based clinical guidelines, economic evaluation, and multidisciplinary collaboration collectively represent essential components for translating biomarker discoveries into meaningful improvements in patient care. As high-throughput multi-omics technologies, artificial intelligence, and precision nutrition continue to evolve, biomarker-guided prediction models are expected to become increasingly sophisticated and clinically applicable. Ultimately, these advances have the potential to transform breast cancer management by enabling more accurate risk prediction, individualized therapeutic decision-making, optimized nutritional interventions, and improved long-term patient outcomes.

## 5 Discussion and future directions

### 5.1 Methodological considerations and systematic limitations

Current nutritional biomarker research in breast cancer is constrained by several interconnected methodological limitations that must be systematically addressed. First, analytical and pre-analytical standardization remains inadequate. Variability in biomarker measurements arises from sample collection timing, fasting status, processing delays, storage temperatures, and inter-platform differences, all of which obscure true associations and reduce reproducibility across studies. Second, the existing evidence base is dominated by single-center retrospective cohorts with limited external validation. Such designs are susceptible to selection bias, confounding, and overfitting, particularly when high-dimensional omics data are analyzed without adequate adjustment. Third, the overwhelming majority of studies have been conducted in Western populations (128), severely limiting generalizability to Asian, African, and other ethnic groups with distinct dietary patterns, body compositions, and genetic backgrounds. Fourth, single biomarkers demonstrate only modest predictive performance; breast cancer is a multifactorial disease requiring integrated multi-omic signatures to achieve clinically meaningful discrimination. Fifth, and critically, there is a paucity of long-term, randomized controlled trials (RCTs) evaluating whether biomarker-guided nutritional interventions actually improve hard clinical endpoints such as incidence, recurrence, or survival. Without such intervention evidence, the clinical utility of nutritional biomarkers remains confined to risk stratification rather than therapeutic guidance.

### 5.2 Toward precision nutrition in breast cancer

Precision nutrition seeks to tailor dietary interventions according to individual biological characteristics, including genetic background, metabolic phenotype, gut microbiome composition, endocrine status, and lifestyle factors. In breast cancer, this paradigm provides an opportunity to integrate nutritional biomarkers with tumor biology across the full natural history of the disease—from epithelial transformation and ductal carcinoma *in situ* through invasive progression, metastatic dissemination, and treatment-resistant relapse. For example, during early pathogenesis, folate status and DNA methylation patterns influence genomic stability; during localized disease, vitamin D and inflammatory indices modulate tumor microenvironment and immune surveillance; during systemic therapy, insulin resistance and adipokine profiles predict endocrine and chemotherapy resistance; and during survivorship, longitudinal metabolic monitoring may detect recurrence-prone phenotypes.

Genetic variation affecting nutrient metabolism modifies the relationship between nutritional biomarkers and breast cancer prognosis. Polymorphisms in MTHFR, CYP1A1, COMT, and IRS-1 (129, 130) influence individual susceptibility to dietary exposures and endocrine dysregulation. Integrating genetic profiles with nutritional biomarker data may improve risk prediction accuracy while enabling tailored dietary recommendations. The gut microbiome further mediates diet-breast cancer interactions through nutrient metabolism, microbial metabolite production, and immune homeostasis (131). Interindividual variability in microbial metabolism of phytoestrogens (132) may explain inconsistent epidemiological findings and highlights the microbiome as both a biomarker source and a therapeutic target.

To advance precision nutrition, several actionable research directions merit priority. (i) Formulate unified, internationally accepted standards for biomarker sampling, processing, and quantification to ensure reproducibility across laboratories and populations. (ii) Conduct large-scale, multicenter, externally validated prospective cohorts that span diverse ethnicities and dietary cultures, enabling robust model generalizability. (iii) Adopt Mendelian randomization designs (133, 134) to strengthen causal inference regarding nutritional biomarkers and breast cancer outcomes, mitigating confounding and reverse causation inherent in observational studies. (iv) Implement dynamic joint monitoring platforms that combine repeated nutritional biomarker assessment with digital dietary intake recording, wearable physical activity monitors, and clinical outcome tracking to capture longitudinal metabolic trajectories rather than single-timepoint snapshots. (v) Develop biomarker-stratified RCTs in which high-risk metabolic phenotypes are randomized to targeted nutritional interventions versus standard care, thereby generating definitive evidence for precision nutrition efficacy.

It must be explicitly acknowledged that a substantial proportion of the mechanistic evidence synthesized in this review derives from preclinical basic studies, *in vitro* experiments, and animal models rather than from real-world intervention cohorts or prospective clinical trials. While these foundational studies establish biological plausibility, they do not establish clinical efficacy. Consequently, the near-term applicability of precision nutrition strategies in routine oncology practice should not be overestimated. Translating biomarker discoveries into clinically actionable dietary guidelines will require rigorous, long-term prospective validation and biomarker-guided intervention trials before widespread adoption can be recommended.

### 5.3 Integrative approaches and clinical translation

Integrating nutritional and endocrine biomarkers into clinical oncology necessitates a comprehensive multidisciplinary approach involving diverse professionals. Oncologists, nutritionists, endocrinologists, pathologists, and bioinformaticians must collaborate to harness the full potential of biomarker data in the realm of personalized medicine. Crucial to this integration is the development of sophisticated clinical decision support systems that not only incorporate these biomarkers but also align with traditional prognostic criteria. Such tools must be intuitively designed, grounded in rigorous evidence to ensure scientific validity, and seamlessly woven into the fabric of electronic health records to facilitate their adoption within the pressures of modern clinical practice.

Nutritional intervention opens a new direction for breast cancer management and prevention by regulating endocrine pathways. A promising strategy combines dietary energy restriction with omega-3 fatty acids (135), simultaneously intervening in multiple obesity-related endocrine pathways including IGF-I bioavailability, oxidative stress, lipid metabolism, and inflammatory cytokine activity. Research focusing on this dual intervention model in high-risk overweight and obese women aims to determine whether cancer risk can be reduced through modulation of scientifically validated tissue biomarkers. Success in such research may provide safe and effective chemopreventive means for high-risk populations while elucidating the biological mechanisms of breast cancer occurrence and development.

As we anticipate future research endeavors, a priority should be placed on randomized controlled trials that precisely target biomarker-guided nutritional interventions within well-defined patient cohorts (136). These trials must encompass meticulous assessment of biomarkers across various intervals, aiming to uncover predictive factors of response and to unravel the mechanisms of action. Furthermore, implementation science must be leveraged to identify and surmount barriers hindering the adoption of biomarker-informed practices across diverse healthcare ecosystems [139[. With the accrual of evidence, updates to clinical guidelines are imperative to encapsulate the pivotal role of nutritional and endocrine biomarkers in breast cancer risk assessment and subsequent management strategies (**Figure 5**).

**Figure 5:**
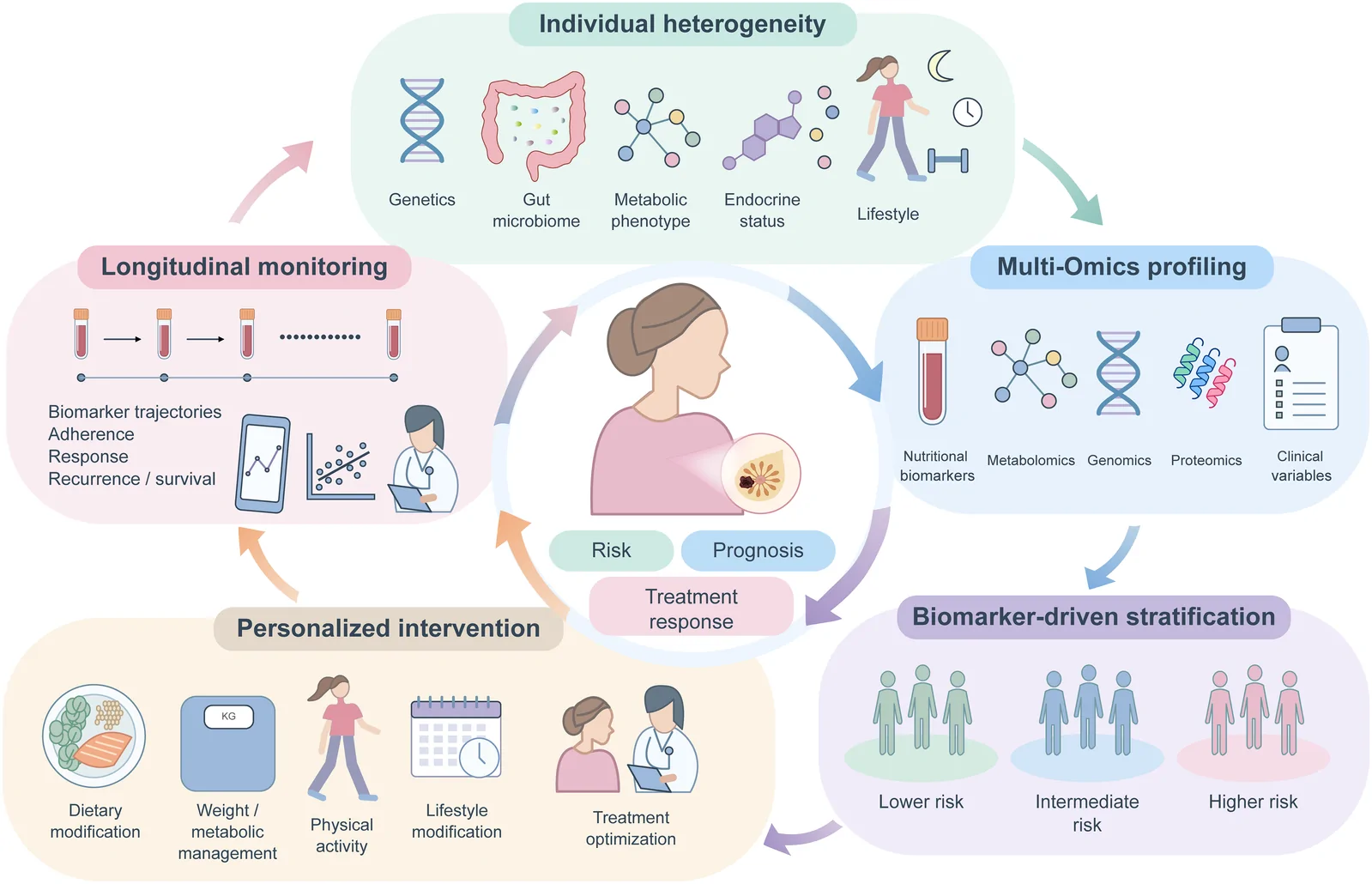
Closed-loop precision nutrition framework integrating individual heterogeneity (genetics, microbiome), multi-omic profiling, biomarker-driven stratification, personalized lifestyle interventions, and longitudinal monitoring to dynamically refine breast cancer care.

The transition of nutritional biomarker research from bench to bedside also necessitates a focus on educational development across the healthcare continuum. Programmatic changes should be instituted within medical education to include training in nutritional sciences and their relevance to oncological practice (137). Furthermore, patients must be equipped with access to credible information and support networks to comprehend the significance of nutrition in cancer care and to facilitate the implementation of evidence-based dietary modifications. Digital health platforms, such as mobile applications and telemedicine services, hold promise as conduits for the delivery of individualized nutrition interventions and as levers for catalyzing large-scale behavioral change (138). Finally, the healthcare infrastructure must accommodate sustainable ways to incorporate nutrition services into the typical oncology care regimen, ensuring universal access to nutritional assessment and intervention (139, 140). These integrated approaches are crucial in realizing the transformative potential of nutritional biomarkers in enhancing breast cancer outcomes and in providing a more personalized, effective care approach.

## 6 Conclusion

This review synthesizes contemporary evidence regarding nutritional biomarkers and associated endocrine mechanisms governing breast cancer risk and prognosis across the disease continuum—from early pathogenesis through treatment and survivorship. Dietary components engage in intricate crosstalk with metabolic and hormonal pathways, yielding critical insights for breast cancer prevention and modulation of tumor progression. Nutritional biomarkers serve as objective readouts capturing these complex biological interactions, representing promising tools to refine risk stratification and prognostic evaluation in clinical practice. Incorporating nutritional and endocrine biomarkers into predictive models paves the way for personalized breast cancer management. Validated models that discriminate high-risk populations reinforce preventive measures and may improve treatment responses among diagnosed patients. Future research must prioritize analytical standardization, large-scale prospective and intervention studies across diverse populations, and integrated multi-omic frameworks to strengthen the evidence base for biomarker-targeted intervention strategies. As consistent empirical evidence accumulates, these biomarkers carry substantial potential to transform routine breast cancer care, facilitating effective individualized risk reduction and refined therapeutic optimization.
